# Soot and the city: Evaluating the impacts of Clean Heat policies on indoor/outdoor air quality in New York City apartments

**DOI:** 10.1371/journal.pone.0199783

**Published:** 2018-06-28

**Authors:** Carlos F. Gould, Steven N. Chillrud, Douglas Phillips, Matthew S. Perzanowski, Diana Hernández

**Affiliations:** 1 Department of Environmental Health Science, Mailman School of Public Health of Columbia University, New York, New York, United States of America; 2 Lamont-Doherty Columbia Earth Observatory of Columbia University, Palisades, New York, United States of America; 3 Department of Sociomedical Sciences, Mailman School of Public Health of Columbia University, New York, New York, United States of America; Ball State University, UNITED STATES

## Abstract

New York City has had a long history of implementing local policies to reduce air pollution. Enacted as a part of PlaNYC, the Clean Heat policies aim to lower wintertime ambient air pollution by phasing out dirty No. 6 heating fuel oil and transitioning to comparatively cleaner No. 4, No. 2, or natural gas. This study evaluates the impacts of policies on ambient air pollution and, given that people spend the majority of their time inside, importantly, indoor air pollution. Using a natural experiment, we evaluate the effects of the policies by measuring average two-week levels of indoor and outdoor black carbon (BC) and fine particulate matter (PM_2.5_) in 48 upper Manhattan apartments in successive winter heating seasons before and after mandated fuel transition. We failed to observe systematic improvements in indoor BC and PM_2.5_ concentrations in follow-up. However, outdoor levels of PM_2.5_ did improve, with statistical differences observed among buildings converting to the cleanest fuels. Non-statistical improvements were observed for outdoor BC. However, when accounting for meteorological differences, apartment characteristics, and behavioral patterns that may have influenced air pollution measurements, these differences were not significant. The study results have important policy and equity implications considering the differential improvements in air quality by conversion to No. 4 oil as compared to the cleaner No. 2 oil and natural gas.

## Introduction

Exposure to air pollution remains one of the most significant global health risks today [1]; in 2015 exposure to air pollution was the sixth leading risk factor for deaths in the United States, accounting for an estimated 88,400 deaths (3% of all deaths) and 1.49 million disability-adjusted life years [2]. In response, air pollution has been the target of many policy efforts in the United States over the past 50 years. By focusing on fuel standards and emission sources for air pollutants like black carbon (BC), sulfur dioxide, and lead, policies have had notable success in lowering ambient air pollution [3–6]. Robust epidemiologic studies show positive associations between ambient concentrations of particulate matter (PM) and excess morbidity and mortality, suggesting that air pollution improvements may yield significant public health benefits [7–9]. Since people spend a majority of their time indoors [10–12], however, the relationship between ambient concentrations and personal exposure is not always well correlated [13–15]. Importantly, the effects of policies aimed to lower ambient air pollution on indoor air quality have not been properly characterized.

Unlike many cities in the United States and around the world that are most affected by traffic-related air pollution, residential, commercial, and institutional heating systems in New York City (NYC) are estimated to release 50% more fine particulate matter (PM_2.5_) than vehicles [16]. In 2009, residential buildings burning No. 6 oil (5,500 boilers) and No. 4 (3,500 boilers), comprising only about 1% of all the city’s buildings, accounted for an estimated 86% of NYC’s *heating-related* PM_2.5_ emissions [16]. Burning No. 6 and No. 4 oils produces significantly more air pollution than cleaner fuel sources like No. 2 oil or natural gas because higher concentrations of sulfur and other contaminants (metals) lead to greater incomplete combustion [16]. As a result, NYC air pollution exhibits temporal and geographic variability in emission rates, with increases during the wintertime heating season and increased pollution associated with residual fuel boiler density [17,18].

Implemented in 2012, the Clean Heat policies targeted local emissions driving high wintertime neighborhood air pollution—specifically, PM_2.5_, BC, sulfur, and nickel—as a part of PlaNYC, the city’s sustainability plan of 127 initiatives to improve environmental quality [19,20]. The policies phased out the use of No. 6 oil in residential and commercial boilers, mandating that buildings burning No. 6 oil convert to the comparatively cleaner-burning, lower-sulfur No. 4 oil (a mixture of No. 6 and No. 2 oil), No. 2 oil, or natural gas. At study start in December 2013, few buildings had transitioned from No. 6 oil to a cleaner fuel, while by the beginning of the second monitoring period a year later in December 2014 still 4,888 buildings were burning No. 6 oil while another 3,160 were burning No. 4 oil [21]. By the end of 2015 after the conclusion of the study, only 21 buildings were burning No. 6 oil but still 3,557 were burning No. 4 oil [22]. In total, between 2012 and 2015, the policies reported 99.8% compliance in residential buildings [23]. Ambient air quality policies such as Clean Heat can have dramatic health impacts as per the epidemiologic evidence for improved population health with decreased levels of exposure [24–26]. Since people spend the majority of their time indoors, mostly at home, during the winter the impacts of policies on indoor air pollutions is of great interest and importance. Given NYC’s size and influence, this regulation marks one of the largest and most comprehensive pieces of public health, environmental, and energy policy in the past decade.

The present study assesses the impact of the Clean Heat policies on indoor and outdoor PM_2.5_ and BC in apartments located in northern Manhattan phasing out No. 6 oil in the 2013–2014 and 2014–2015 heating seasons. In doing so, we take advantage of a unique opportunity to evaluate the actual impacts of a suite of measures meant to improve NYC’s ambient air pollution. We hypothesized that apartments in residential buildings phasing out No. 6 would lower PM_2.5_ and BC during the heating season, and that buildings converting to cleaner fuels (No. 2 or natural gas) would have incrementally more improvements than those converting to a dirtier fuel (No. 4). Furthermore, we conduct exploratory analyses of apartment and building characteristics and behavioral patterns that influence indoor air pollution and the potential impacts of policies reducing ambient air pollution on personal exposure indoors.

## Methods

### Building recruitment

The study team identified 269 potential buildings in northern Manhattan (north of 100^th^ street) with a mandatory conversion date from No. 6 oil to a cleaner fuel source (No. 4 fuel, No. 2 fuel, or natural gas) between the 2013–14 heating season and the 2014–15 heating season using a publicly available list of buildings implicated by the clean heat laws [22]. A final list of 250 buildings was established by 1) eliminating buildings with less than 5 five stories (a requirement to differentiate between BC sources like vehicle exhaust emissions and from heating oil between apartments on lower and upper floors) and 2) limiting to buildings only one boiler to maintain consistency between target buildings. Indoor smokers were not eligible for study participation.

In total, the air quality in 48 apartments in 27 buildings was monitored in both the 2013–14 (sampling between December 2013-April 2014) and 2014–15 (sampling between December 2014-April 2015) heating seasons. Study participants were enrolled in the study via door-to-door recruitment methods. Exposure assessments were conducted in an evenly split number of upper and lower floor apartments. Efforts were made to monitor enrolled apartments over the same time-period between pre- and post-conversion. In the 2014–15 heating season, 15 buildings with 27 apartments were burning No. 4 oil, 6 buildings with 11 apartments were burning No. 2 oil, and 6 buildings with 10 apartments were burning natural gas. While 60 apartments evenly split between upper and lower floors were originally enrolled, 12 were lost between the two measurement periods due to failure of building conversion to a cleaner fuel (n = 6), refusal to participate in follow-up monitoring (n = 3), a landlord that objected to measurements during the first monitoring phase (n = 2), and a tenant that relocated between the assessment periods (n = 1) (Table A in S1 File). Table B in S1 File compares apartments lost to follow-up to those in the study sample in terms of participant characteristics, behavioral patterns, and pre-conversion indoor and outdoor air pollution; though it is a small sample size for comparisons, there are no systematic differences observed. Two outdoor samples (one pre-conversion sample and one post-conversion in different apartments) were lost due to instrument mechanical failure and were thus removed from statistical analyses.

The full questionnaire administered to study participants to collect basic demographic, apartment, and behavioral information is available in the S2 File.

### PM_2.5_ and BC measurements

Indoor (e.g., living room) and outdoor (via tube passing outside) PM_2.5_ and BC measurements were collected in each apartment for two weeks during the 2013–14 heating season prior to heating oil conversion and for two weeks during the next heating season between 2014–15. The PM_2.5_ filters collected continuously from inside and outside apartments at 1.5 L/min for 14 consecutive days using a triplex cyclone (BGI, Waltham, MA) with a PM_2.5_ cut point. This sampling time period aimed to average two 6–7 day weather cycles typical of NYC, and thus provide an estimate of a seasonal average that is less affected by the potential for short-term extreme cold or warm days. The filters were weighed pre- and post-sampling in a specialized weighing chamber to determine net mass of PM_2.5_ collected onto the filters. The methods above have been used in several NYC studies carried out at Columbia University [6,17,18]. BC concentrations were determined from the Teflon filters of PM_2.5_. Measurements of BC were made using a multi-wave length optical absorption technique on filters of PM_2.5_ following a published procedure [27].

### Reference data

Reference sites for BC and PM_2.5_ data during the sampling period provide contextualization for longer-term trends and impacts from weather patterns. The reference site for the BC measurements, made by an aethalometer (Magee Scientific), was located at West Harlem Environmental Action, Inc. The reference site for PM_2.5_ was located at the New York State Department of Environmental Conservation PM_2.5_ monitoring site in northern Manhattan [28]. In conjunction with meteorological data from the NOAA Central Park site, these data were used to help characterize the residential changes attributable to weather patterns by controlling for variables like temperature, wind speed, and to provide ancillary data on regional changes and additional sources of BC for each building site. Fig A in S1 File shows the mean central site temperature and wind speed during both sampling periods for all apartments. In addition, Table C in S1 File shows descriptive statistics of indoor temperature during both sampling periods for all apartments. Continuous BC and PM_2.5_ data sources at reference sites were used to contextualize observed indoor and outdoor air pollution levels into the overall levels seen throughout the city at the time of sample collection (Fig B in S1 File).

Data from the New York City’s “Spot the Soot” program associated with the Clean Heat policies were used to describe study building characteristics and provide indications of neighborhood-level trends in heating fuel conversion [22].

### Statistical analyses of air pollution

#### Raw data

Summary statistics for wintertime indoor and outdoor BC and PM_2.5_ concentrations were calculated before and after fuel conversion for all apartments, as well as among apartment subgroups by fuel conversion type and building floor category. Two-sided t-tests were used to determine if differences before and after fuel conversion were significant.

#### Corrected data

Since meteorological conditions have a large and complex impact on indoor and outdoor air pollution [17,29,30], two different methods were used to correct for meteorological differences during pre-conversion and post-conversion sampling.

1. Outdoor air pollution measurements were corrected in statistical analyses utilizing reference site measurements by dividing the observed apartment measurement by a correction factor (reference site mean for the same time periods as the sample divided by the reference site’s seasonal mean). This approach assumes that the concentration at the fixed site monitor is primarily impacted by meteorological conditions as well as the overall regional changes in air quality, but that its levels are not impacted directly by the policy change (i.e., that the monitor was not located on a building in or adjacent to buildings that converted fuel type).
2. A subset analysis was done for apartments which had the most similar
  - mean wind speeds during the two-week sampling periods (within 0.29 m/s (n = 24)) since large differences in wind speed affect local air pollution dispersion.
  - mean ambient temperatures during the two-week sampling periods (within 7.59°C (n = 24) and within 2.73°C (n = 12)).

All analyses described for the raw data were repeated for the corrected data.

#### Secondary analysis of air quality changes and impacts on air quality

To more robustly investigate the relationships between observed indoor and outdoor BC and PM_2.5_, meteorological factors, apartment and building characteristics, and behavioral patterns, we conducted several secondary analyses (rationale and hypotheses available in S1 File):

1. **Meteorological factors:** In addition to analysis in apartment subsets with similar meteorological factors in both monitoring periods, we include wind speed and temperature in univariable and multivariable regressions
2. **Apartment characteristics:** Linear regression models controlling for fuel type and floor category were used for both indoor and outdoor air quality to determine the importance of these potential covariates on observed air quality. We additionally evaluated multivariable regressions with predictors that included apartment characteristics as independent variables and before-after air pollution changes for indoor and outdoor BC and PM_2.5_ as the dependent variable. Then, we conducted subset analyses on specific apartment characteristics.
3. **Behavioral patterns:** Analyses of air pollution differences before and after the fuel transition around potential covariates were conducted utilizing descriptive statistics of apartment behavioral patterns (e.g., window opening, report of secondhand smoke in apartment).
4. **Building characteristics:** Univariable and multivariable regression models controlling for building age, boiler capacity, and boiler age were utilized to examine the individual and joint relationships between building characteristics and before and after indoor air pollution, as well as air pollution changes before and after fuel conversion.
5. **I/O ratios:** Indoor/outdoor air pollution ratios were assessed to determine the relative contributions of emissions sources.

### Ethical considerations

This study was reviewed and approved prior to initiation of the research by the Institutional Review Board (IRB) at the Columbia University Medical Center. All study participants provided written consent.

## Results

### Apartment descriptive statistics

Descriptive statistics of study participants are shown in Table 1 and further descriptive statistics of study participant behavior and apartment conditions are included in Table 2. Respondents reported spending the majority of their time indoors, especially during the winter. While indoor smoking was an exclusion criterion for participation and expectedly low in the sample, more than half reported burning candles or incense in their apartments. Furthermore, about half of respondents reported experiencing second-hand smoke from outside enter their apartment either monthly or more frequently and a majority of respondents reported opening their windows because their apartment was too hot during the heating season. Table 3 shows descriptive statistics of study building characteristics.

**Table 1:** Sample characteristics (at baseline).

	Mean / Percent	N	SD	Min	Max	Received Question^a^
*Gender*						48
Male	35%	17				
Female	65%	31				
*Age (years)*	45.77		15.30	23	85	48
*Race/Ethnicity*						48
Non-Hispanic White	52%	25				
Non-Hispanic Black or African-American	10%	5				
Hispanic or Latino	27%	13				
Asian or Pacific Islander	8%	4				
Multiracial	2%	1				
*Education*						48
Elementary/Primary	2%	1				
High School/Secondary	15%	7				
2-Year Community College or Vocational	8%	4				
4-Year College or Greater	75%	36				
*Employment Status (Unemployed)*	10%	5				48
*Household Income*						48
Below $29,999	29%	14				
$30,000-$49,999	10%	5				
Above $50,000	56%	27				
*Number of People Living in the Household*	2.75		1.31	1	7	48
*Household with Children (under 18)*	35%	17				48
*Housing Tenure (Renters)*	94%	45				48
*Years Lived at Residence*						26
< 1	27%	7				
1–2	23%	6				
2–5	23%	6				
> 5	27%	7				
*Monthly Rent ($)*	1487		650	516	2700	25
*Housing hardship (e*.*g*., *making rent*, *homelessness)*	7%	3				25

^a^ Some participants did not receive all questions and some did not answer all questions.

**Table 2:** Behavioral descriptive statistics of sample (at baseline, N = 48).

Question	Percent	N
*During the winter months*, *during the week*, *meaning Monday through Friday*, *how much time do you spend inside your home during the day?*		
Most or all of the day inside	38%	18
Little or only some of the day inside	62%	30
*Does this change depending on the season?*		
More time outside in the summer	67%	32
No	33%	16
*During the winter*, *during the weekend*, *meaning Saturday and Sunday*, *how much time do you spend inside your home during the day?*		
Most or all of the day inside	44%	21
Little or only some of the day inside	56%	27
*Does anyone in the home burn candles*, *incense*, *or anything like that indoors?*		
Yes	63%	30
No	37%	18
*How often would you say those things are burned indoors?*		
Once a week or more frequently	63%	18
Less than once a week	37%	12
*Do any members of your household smoke indoors*		
Yes	10%	5
No	90%	43
*In the last 12 months*, *how often has second-hand tobacco smoke entered inside your home from somewhere else in or around the building?*		
Monthly or more frequently	42%	20
Never or less frequently than monthly	52%	25
*Do you ever open windows because your housing unit was too hot?*		
Yes	73%	35
No	25%	12
*About how often did you open windows because it was too hot?*		
Once a week or more frequently	63%	22
Less than once a week	37%	13

**Table 3:** Building characteristics.

	All Study Buildings (n = 24)^a^	Dirty Fuel (n = 13)	Clean Fuel (n = 11)
*Mean Building Age (Years)*	92	93	92
*Mean Building Square Feet*	60727	61691	59985
*Mean Total Units*	60	59	60
*Mean Number of Floors*	6.0	6.1	6.0
*Mean Boiler Age (Years)*	26	27	25
<10 Years	26%	20%	31%
11–20 Years	17%	20%	15%
21–30 Years	48%	50%	46%
>31 Years	4%	10%	0%
*Mean Boiler Capacity* *(Gross BTU)*	4.8	4.9	4.7

^a^ Three buildings have no data because of irreconcilable differences in addresses between NYC Building Database and Study Apartment Addresses.

### Air pollution measurements

#### Fine particulate matter

While indoor PM_2.5_ stayed the same before and after in follow-up at 12.8 μg/m^3^ (p = 0.99), outdoor air pollution fell from 8.96 to 8.08 μg/m^3^ (p = 0.08) (Table 4). When looking only at apartments switching to clean fuels (No. 2 or natural gas), outdoor air pollution fell significantly after fuel conversion (p = 0.01). No statistically significant reductions were observed in apartments switching to dirtier fuels. While changes in indoor PM_2.5_ largely diverged by apartment floor type, where levels fell in upper floor apartments and rose in lower floor apartments, outdoor PM_2.5_ fell post-conversion on average.

**Table 4:** Observed PM_2.5_ measurements (raw data).

	All Households (#6 to any fuel)	#6 to #2 or gas (cleaner fuels)	#6 to #4 (dirtier fuel)
*Indoor measures (μg/m*^*3*^*)*	*Mean ± SD (n)*	*Mean ± SD (n)*	*Mean ± SD (n)*
Pre-conversion	12.8 ± 7.93 (48)	12.9 ± 8.86 (21)	12.8 ± 7.30 (27)
Post-conversion	12.8 ± 11.9 (48)	12.2 ± 12.3 (21)	13.4 ± 11.71 (27)
p-value^a^	0.99	0.50	0.40
Upper floor			
Pre-conversion	12.9 ± 8.58 (25)	14.5 ± 11.1 (11)	11.6 ± 6.04 (14)
Post-conversion	11.8 ± 10.6 (25)	13.5 ± 15.3 (11)	10.4 ± 4.69 (14)
p-value	0.48	0.65	0.60
Lower floor			
Pre-conversion	12.8 ± 7.36 (23)	11.0 ± 5.44 (10)	14.1 ± 8.51 (13)
Post-conversion	14.0 ± 13.2 (23)	10.7 ± 8.31 (10)	16.5 ± 15.9 (13)
p-value	0.68	0.87	0.63
*Outdoor measures (μg/m*^*3*^*)*			
Pre-conversion	8.96 ± 3.57 (47)	8.85 ± 1.78 (21)	9.05 ± 4.58 (26)
Post-conversion	8.08 ± 1.98 (47)	7.67 ± 1.71 (21)	8.42 ± 2.16 (26)
p-value	0.08	**0.01**	0.44
Upper floor			
Pre-conversion	9.46 ± 4.53 (25)	8.75 ± 1.50 (11)	10.0 ± 5.95 (14)
Post-conversion	8.06 ± 2.00 (25)	7.39 ± 1.52 (11)	8.58 ± 2.22 (14)
p-value	0.11	**0.03**	0.35
Lower floor			
Pre-conversion	8.40 ± 2.59 (22)	8.97 ± 2.12 (10)	7.92 ± 1.77 (12)
Post-conversion	8.11 ± 2.01 (22)	7.97 ± 1.93 (10)	8.23 ± 2.15 (12)
p-value	0.47	0.19	0.64

^a^ P-values refer to paired t-tests comparing pre- and post-conversion measurements.

#### Black carbon

Table 5 shows that indoor black carbon levels rose in follow-up, though the differences are not statistically significant (p = 0.30). In contrast, outdoor black carbon levels fell in follow-up (p = 0.22) and more so in buildings converting to cleaner fuels than dirtier fuels, but the difference between the changes in the two groups is not statistically significant (p = 0.16; Welch Two Sample t-test). Lower floor apartments had higher levels of indoor black carbon than upper floor apartments, however the difference is not statistically significant before or after the transition (Before: p = 0.12; After: p = 0.32; Welch Two Sample t-test).

**Table 5:** Observed BC measurements (raw data).

	All Households (#6 to any fuel)	#6 to #2 or gas (cleaner fuels)	#6 to #4 (dirtier fuel)
*Indoor measures (μg/m*^*3*^*)*	*Mean ± SD (n)*	*Mean ± SD (n)*	*Mean ± SD (n)*
Pre-conversion	1.52 ± 0.75 (48)	1.39 ± 0.43 (21)	1.62 ± 0.93 (27)
Post-conversion	1.68 ± 0.87 (48)	1.59 ± 0.61 (21)	1.76 ± 1.04 (27)
p-value ^a^	0.30	0.10	0.62
Upper floor			
Pre-conversion	1.35 ± 0.52 (25)	1.30 ± 0.38 (11)	1.40 ± 0.63 (14)
Post-conversion	1.56 ± 0.60 (25)	1.40 ± 0.43 (11)	1.68 ± 0.70 (14)
p-value	0.15	0.52	0.22
Lower floor			
Pre-conversion	1.70 ± 0.92 (23)	1.48 ± 0.46 (10)	1.86 ± 1.14 (13)
Post-conversion	1.82 ± 1.09 (23)	1.80 ± 0.72 (10)	1.84 ± 1.34 (13)
p-value	0.69	0.10	0.97
*Outdoor measures (μg/m*^*3*^*)*			
Pre-conversion	1.33 ± 0.38 (47)	1.39 ± 0.37 (21)	1.28 ± 0.39 (26)
Post-conversion	1.28 ± 0.32 (47)	1.27 ± 0.33 (21)	1.28 ± 0.31 (26)
p-value	0.22	0.16	0.83
Upper floor			
Pre-conversion	1.35 ± 0.40 (25)	1.37 ± 0.36 (11)	1.34 ± 0.43 (14)
Post-conversion	1.28 ± 0.33 (25)	1.26 ± 0.36 (11)	1.29 ± 0.32 (14)
p-value	0.21	0.25	0.56
Lower floor			
Pre-conversion	1.31 ± 0.38 (22)	1.41 ± 0.40 (11)	1.22 ± 0.35 (12)
Post-conversion	1.27 ± 0.31 (22)	1.29 ± 0.32 (10)	1.26 ± 0.31 (12)
p-value	0.62	0.41	0.76

^a^ P-values refer to paired t-tests comparing pre- and post-conversion measurements.

#### Corrected outdoor pollutant measures

After correction using reference site data, reduction in outdoor PM_2.5_ was significant when including all apartments (p = 0.04) and also among dirty fuel apartments (p = 0.03) (Table 6). Reductions observed in outdoor BC in all apartments were not statistically significant (p = 0.08). Like outdoor PM_2.5_, outdoor BC measures fell among dirty fuel apartments (p = 0.05). Raw outdoor PM_2.5_ reductions in clean fuel apartment were no longer statistically significant after correction.

**Table 6:** Corrected outdoor pollutant measures ^b^.

	All Households (#6 to any fuel)	#6 to #2 or gas (cleaner fuels)	#6 to #4 (dirtier fuel)
*PM2*.*5 (μg/m*^*3*^*)*	*Mean ± SD (n)*	*Mean ± SD (n)*	*Mean ± SD (n)*
Pre-conversion	10.1 ± 5.35 (47)	8.59 ± 2.22 (21)	11.3 ± 6.74 (26)
Post-conversion	8.12 ± 3.37 (47)	8.30 ± 3.61 (21)	7.98 ± 3.22 (26)
p-value^a^	**0.04**	0.77	**0.03**
Upper floor			
Pre-conversion	10.7 ± 6.89 (25)	8.58 ± 2.36 (11)	12.3 ± 8.77 (14)
Post-conversion	8.05 ± 3.19 (25)	8.05 ± 3.42 (11)	8.06 ± 3.14 (14)
p-value	0.07	0.69	0.08
Lower floor			
Pre-conversion	9.39 ± 2.73 (22)	8.59 ± 2.17 (10)	10.1 ± 3.05 (12)
Post-conversion	8.20 ± 3.63 (22)	8.59 ± 3.97 (10)	7.88 ± 3.45 (12)
p-value	0.33	0.99	0.17
*BC (μg/m*^*3*^*)*	* *	* *	* *
Pre-conversion	1.38 ± 0.37 (47)	1.28 ± 0.22 (21)	1.46 ± 0.45 (26)
Post-conversion	1.29 ± 0.32 (47)	1.27 ± 0.28 (21)	1.30 ± 0.35 (26)
p-value	0.08	0.80	0.05
Upper floor			
Pre-conversion	1.40 ± 0.41 (25)	1.27 ± 0.22 (11)	1.50 ± 0.50 (25)
Post-conversion	1.29 ± 0.35 (25)	1.23 ± 0.31 (11)	1.34 ± 0.38 (25)
p-value	0.14	0.67	0.16
Lower floor			
Pre-conversion	1.37 ± 0.33 (22)	1.30 ± 0.22 (10)	1.42 ± 0.41 (22)
Post-conversion	1.28 ± 0.30 (22)	1.30 ± 0.27 (10)	1.26 ± 0.33 (22)
p-value	0.31	0.98	0.22

^a^ P-values refer to paired t-tests comparing pre- and post-conversion measurements.

^b^ Outdoor measurements are adjusted using central site measurements by dividing observed measurement by a correction factor of center site mean over seasonal mean.

### Secondary analyses

We examined the relationships between observed indoor and outdoor air pollution to assess the independence of these measures. Univariable linear regressions between indoor and outdoor air pollution before and after fuel transition were largely not statistically significant, which suggests mediation of the relationship between indoor and outdoor air pollution by external factors (Table D in S1 File). We conducted secondary analyses to investigate potential external factors affecting the indoor-outdoor air pollution relationship. Such factors could mediate the impacts of Clean Heat policies on indoor air pollution.

#### Meteorological factors

Given the relationship between indoor and outdoor air pollution and local dispersion patterns, local meteorological factors like wind speed and outdoor temperature may be important factors to observed air pollution measurements [31,32]. While these subsets were challenged by small numbers of apartments, they offer some indication as to the potential for confounding by apartment characteristics.

1. **Wind Speed**: Simple linear regressions showed a significant relationship between average daily wind speed and observed outdoor air pollution during both heating seasons (Table E in S1 File). We therefore limited analysis to apartments with absolute changes in average daily wind speed below the median (0.29 m/s) (n = 24) (Table F in S1 File). Outdoor PM_2.5_ fell significantly among dirty fuel by 2.11 μg/m^3^ (p = 0.04) and BC showed no obvious trends.
2. **Outdoor Temperature:** A building’s heating requirements are subject to the outdoor temperature. Absolute changes in average daily temperature were significantly associated with differences in outdoor PM_2.5_ and BC changes in linear regressions (Table G in S1 File). We conducted analyses among apartments with below the absolute median temperature change before and after the fuel transition (7.59 degrees F) (n = 24) and in the lowest quartile (2.72 degrees F) (n = 12). This analysis of raw data reveals no significant differences or trends but outdoor PM_2.5_ measures suggest declines after heating fuel transition (p = 0.06) (Table F in S1 File; Table H in S1 File). The analysis was repeated using corrected outdoor measures (Table I in S1 File); here, PM_2.5_ fell significantly in dirty fuel apartments with similar wind speed after fuel transition (p = 0.04). However, in both analyses there were no consistent trends.
3. **Meteorologically similar apartments**: To consider the effects of both wind speed and temperature together, we conducted an analysis of apartments with both similar temperature and wind speed before and after the fuel transition (n = 12) (Table J in S1 File). Among this small subset, outdoor PM_2.5_ fell significantly after the fuel transition (p = 0.04). Furthermore, indoor PM_2.5_ and lower floor BC levels decreased significantly following the fuel transition (p = 0.04; p = 0.04). Among these apartments, outdoor measurements fell consistently but significant declines were inconsistent.

#### Behavioral patterns

In multivariable regressions controlling for fuel type and floor category, we observe a significant relationship between report of secondhand smoke entering the apartment and indoor PM_2.5_ difference before and after fuel transition (Table K in S1 File). This suggests the importance of analyses accounting for these behavioral patterns in describing indoor and outdoor air pollution.

1. **Opening windows:** Among the 33 apartments reporting to open their windows as a heat-management strategy during the monitoring period, outdoor PM_2.5_ fell significantly by 3.14 μg/m^3^ (p = 0.01) (Table L in S1 File). In addition, indoor PM_2.5_ fell by 2.17 μg/m^3^, a decline that was nearly significant (p = 0.06).
2. **Secondhand smoke report:** Report of secondhand smoke entering an apartment indicates a relationship with outdoor air pollution that could affect measurements. We conducted an analysis of both respondents reporting secondhand smoke entering the home at least monthly or more frequently (n = 20) and those without such report (n = 26) (Table M in S1 File). There were no obvious trends in air pollution observed among either sample.

#### Building characteristics

Simple linear regressions between building characteristics and indoor air pollution show inconsistent trends for the impacts of building age, boiler capacity, and boiler age on indoor PM_2.5_ and BC (Table N in S1 File). Multivariable regressions with all variables also show variable trends, with few significant coefficients (Table O in S1 File).

#### Indoor/Outdoor ratios

Indoor/outdoor ratios for both BC and PM_2.5_ increased after the fuel transition, suggesting an increased burden from indoor emissions sources (Table P in S1 File). Before the fuel transition, BC indoor/outdoor ratios were significantly lower than PM_2.5_ indoor/outdoor ratios (p = 0.02; Welch Two-Sample T-test), but the difference was no longer significant after fuel transition.

## Discussion

In this study, we evaluated indoor and outdoor air pollution in 48 northern Manhattan apartments in two successive winters, before and after building conversion to cleaner heating fuels in compliance with New York City’s Clean Heat policies. While there were few significant changes in PM_2.5_ and BC observed, this is among the first efforts to evaluate the impacts of an air pollution policy on both indoor and outdoor air quality.

Study apartments were at or slightly above the EPA’s National Ambient Air Quality Standards (NAAQS) for annual PM_2.5_ of 12.00 μg/m^3^; in total, 17 apartments had observed indoor PM_2.5_ above this level pre-conversion and 14 post-conversion. Observed PM_2.5_ and BC were comparable to data reported by the New York City Community Air Survey (NYCCAS) over the same time-periods [33,34]. NYCCAS has reported significant wintertime declines for SO_2_ and some reductions for PM_2.5_ and BC [35,36], however, in this study we fail to see these city-wide changes extended to our upper Manhattan study sample. NYCCAS is a strong resource for policy makers and scientists, however, in this study we additionally examine the indoor environment and local air pollution patterns, observing differential effects by indoor and outdoor air pollution and by heating fuel conversion type.

Primary analysis shows that outdoor PM_2.5_ fell significantly among apartments converting to cleaner fuels, largely driven by the reductions of upper floor apartments. Correcting outdoor measures for reference site pollution measurements increased the difference between before and after fuel transition for both PM_2.5_ and BC (PM_2.5_: p = 0.04; BC: p = 0.08). In both cases, upper floor apartments largely observed greater reductions in outdoor air pollution as compared to lower floor apartments, though the difference was not statistically significant. Given that lower floor apartments may have greater exposure to static street emissions sources, like vehicular traffic, greater upper floor reductions suggest that changes from heating fuel transitions may have been the primary drivers of reductions. Furthermore, indoor/outdoor air pollution ratios can suggest the influence of indoor sources of air pollution as opposed to strictly outdoor sources of pollution as mitigated by the permeability of building envelopes and behavioral patterns. Observed indoor/outdoor air pollution ratios suggest that there are some indoor sources of pollution, both BC and PM_2.5_. At the same time, the indoor/outdoor ratios observed in this study are similar to those previously reported in a study of NYC air toxics exposures [6].

We explored potential covariates of indoor-outdoor pollution relationships and determinants of personal exposure to air pollution through regressions and analysis within apartment subsets. While the results of the secondary analyses should be interpreted with caution because of relatively low apartment numbers, small effect sizes, and few significant differences, there is evidence supporting the inclusion of meteorological factors (e.g., wind speed, temperature), behavioral patterns (e.g., window opening), and apartment and building characteristics (e.g., floor type, report of secondhand smoke, building age, boiler age, boiler size) in future studies of indoor and outdoor apartment air pollution. Outdoor air pollution measurements, especially outdoor PM_2.5_, largely fell in post-conversion in secondary analyses, though with few significant differences. Furthermore, secondary analyses yielded few consistent patterns of significant results. At the same time, future evaluations of ambient air pollution policies should recognize that the effects of policies on indoor pollution may not be directly correlated to changes to outdoor air pollution as a result of the covariates studied in these analyses.

### Implications for policy and equity

PlaNYC seeks to improve NYC’s environment through ten (largely infrastructural and physical planning) goals for 2030, including achieving the cleanest air quality of any big city in America [37]. The Clean Heat policies are an important aspect of this effort and, if successful, could have significant implications for public health. Despite improvements since the 1970s, air quality in New York City remains a substantial public health threat and ambient pollution levels are among the highest of large cities in the United States [38]. By offering insights into the actual impacts on indoor and outdoor BC and PM_2.5_, our results have important policy and equity implications.

We did not observe systematic differences between apartments transitioning to cleaner fuels as opposed to No. 4 oil. However, the differences in emissions factors between even this low-sulfur No. 4 oil and the cleaner fuels is marked—with No. 6 oil as a reference, No. 4 oil has an emissions factor 18% lower as compared to No. 2 oil and natural gas which have emissions factors 52% and 84% lower, respectively [20]. Nevertheless, this evaluation of the Clean Heat policies does not fully consider the realities of neighborhood inequalities and observes limited effect on the indoor and outdoor air quality of upper Manhattan apartments.

**Fig 1:**
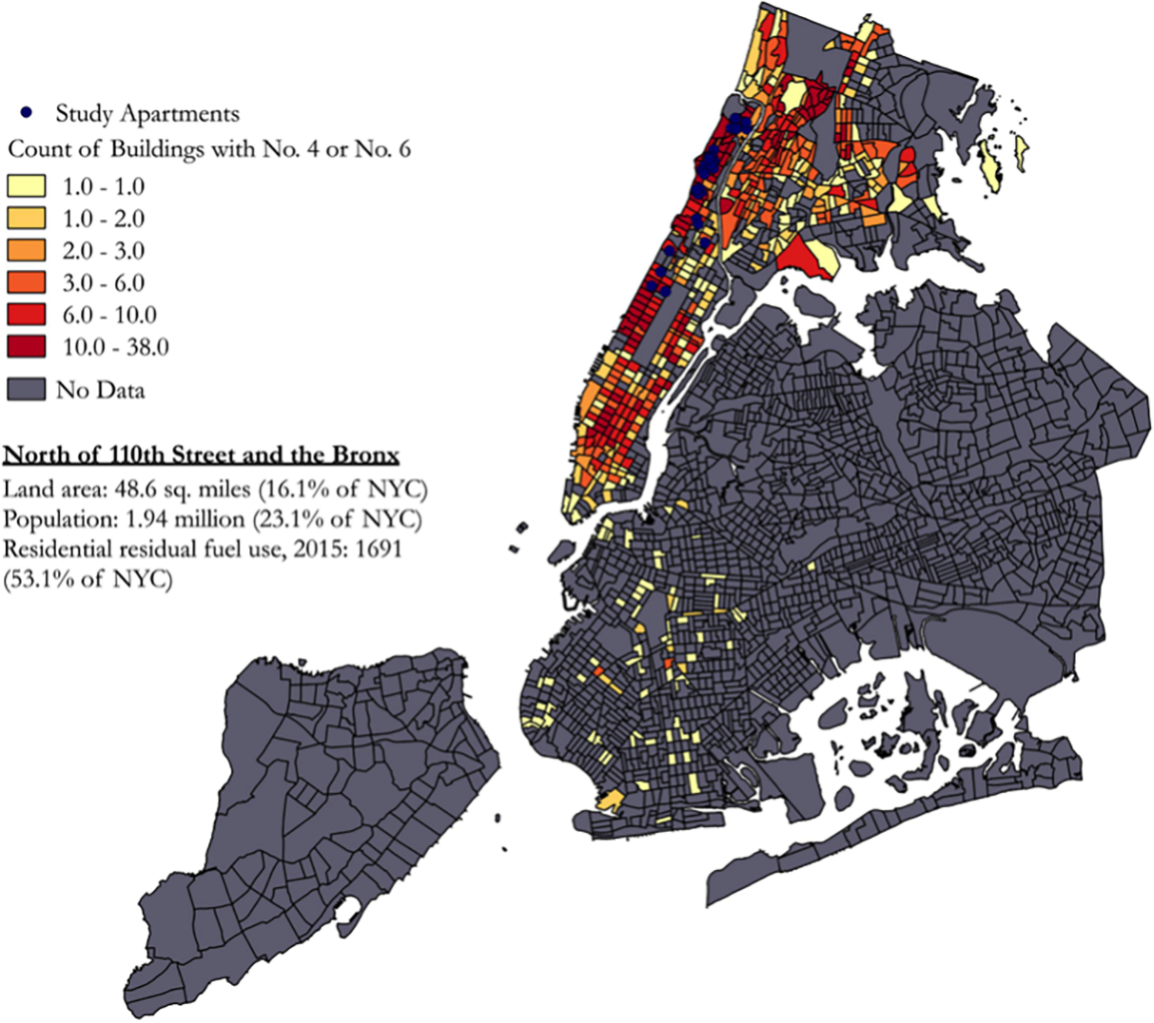
Distribution of study apartments in census tracts with buildings burning No. 4 or No.6 heating oil [22,42].

The transition from No. 6 to No. 4 is not sufficient to achieve appreciable improvements in air quality in an equitable fashion. Compared to the phase out of No. 6, which was achieved within three heating seasons, the complete elimination of No. 4 oil is not due until 2030. Our results provide further support quicker conversion cleaner fuels [23]. Attention to equity is also merited when considering the association between the distribution of clean versus dirty fuels and the socioeconomic status of buildings and neighborhoods. Data from “Spot the Soot” show significant clustering of buildings that converted to No. 4 heating oil as opposed to a cleaner fuel in poorer neighborhoods, including those in our study and nearby (e.g., Northeast Bronx: 52.78%, Washington Heights–Inwood: 45.38%), as illustrated in Fig 1 [22]. Previous efforts to model the effects of the Clean Heat policies on air pollution suggest that the greatest emissions reductions and neighborhood-level health benefits will be obtained in more affluent, high-density areas of Manhattan [39]. The disparate rate of conversion to the cleanest heating fuels might further expose disadvantaged communities to higher air pollution and related health risks, a trend in environmental health disparities observed throughout U.S. cities [40]. Here, we further advocate for the recognition of environmental justice concerns in the indoor environment [41]. Additional supports to lower income buildings and a more aggressive timeline to phase out No. 4 oil are warranted in order to more effectively address these disparities for the sake of air quality and equity.

### Limitations

This study uniquely takes advantage of a natural experiment, observing apartments before and after policy changes. However, despite the richness and rigor of the data collected, the sample size remains small. Although with a larger sample size we may have had sufficient power to detect effects, data collection was encumbered by the time-sensitive nature of this project, especially the collection of baseline data before heating oil conversions. While the apartments included in this analysis had transitioned from No. 6 oil to a cleaner option between the two monitoring periods, the activities and heating oils of surrounding buildings were unknown. In this study we conduct an *interim* investigation of the Clean Heat policy, since it came into effect in mid-2012 and our data collection first occurred during the 2013–2014 heating season. We may expect that the majority of buildings in northern Manhattan by the follow-up monitoring period were no longer using No. 6 oil given strong reported compliance with the Clean Heat policies, however it is possible that surrounding buildings were either (1) already using cleaner fuels in the first monitoring period or (2) continued to use No. 6 oil. In fact, four buildings accounting for six apartments (10% of the original sample) were lost during study follow-up because their buildings were still burning No. 6 and therefore not legally compliant with the policies. This may partially explain the differences observed between our data, where we do not systematically see significant differences, and those reported by NYCCAS, which account for the entirety of the fuel transition process over a five-year timeline. Furthermore, while PM_2.5_ and BC are crucial pollutants with relevant public health outcomes. However, the emissions reductions targeted in the Clean Heat policies also include other pollutants not measured in this study, namely SO_2_ and nickel.

While we attempted to compensate for several factors in secondary analyses, it is possible that our measurements remain confounded. We use comparisons among subsets of apartments with similar before and after average daily temperature to estimate heating requirements; however, we have no information on the volume of fuel used during each monitoring period so we are not able to approximate expected emissions (or reductions) based on the fuel transition. Furthermore, while we hypothesize that air pollution impacts from heating oils are localized, apartment measurements are likely influenced by the surrounding collection of buildings. Air pollution from heating oil emits from chimneys on top of buildings, which could affect downwind neighboring buildings. Similarly, the correction factors applied to outdoor air pollution measurements account for seasonal trends but they fail to consider other shifts between pre- and post-conversion that influence air pollution (e.g., changes in vehicular traffic between years due, in part, to the proliferation of car share services). In addition, unmeasured differences in the built environment between apartments in heating oil categories, like floor type (e.g., carpet, wooden) as it pertains to the resuspension of particulate matter, may have affected results [43,44]. Behavioral patterns may have changed between pre- and post-conversion, too.

## Conclusion

In this study of 48 northern Manhattan apartments, we characterize indoor and outdoor BC and PM_2.5_ in two-week wintertime monitoring periods a year apart to evaluate the Clean Heat policies targeted at improving NYC’s ambient air quality. Primary analysis shows significant outdoor PM_2.5_ reductions among apartments transitioning to cleaner fuels. We carefully integrate meteorological conditions, participant behavioral patterns, and apartment characteristics into further secondary analysis, with evidence suggesting these characteristics may modify the relationships between indoor and outdoor air pollution and personal exposure. Notably, we observe greater reductions in air pollution among apartments converting to cleaner fuels, suggesting that more rapid elimination of No. 4 oil could have significant public health benefits, especially in low socioeconomic status neighborhoods where buildings may still be largely using this dirty fuel.

## Supporting information

S1 File Supporting information on methods, background data, and elaboration of supplemental and sensitivity analyses. (DOCX)

S2 File Full questionnaire as administered to study participants to collect demographic, apartment, and behavioral characteristics. (DOCX)
