# Correction: Soot and the city: Evaluating the impacts of Clean Heat policies on indoor/outdoor air quality in New York City apartments

**DOI:** 10.1371/journal.pone.0202229

**Published:** 2018-08-07

**Authors:** Carlos F. Gould, Steven N. Chillrud, Douglas Phillips, Matthew S. Perzanowski, Diana Hernández

The following information is missing from the Funding section: This study was supported by National Institute of Health grant S10OD016219.
