# Supplementary material for: Soot and the city: Evaluating the impacts of Clean Heat policies on indoor/outdoor air quality in New York City apartments
Source: PLoS One. 2018 Jun 28;13(6):e0199783. doi: 10.1371/journal.pone.0199783 (PMC6023219; doi:10.1371/journal.pone.0199783)
Supplement: S1 File — (DOCX) [file pone.0199783.s001.docx]

**Supporting information**

**Title:** Soot and the City: Evaluating the impacts of Clean Heat Policies on indoor/outdoor air quality in New York City apartments

**Authors:** Carlos F. Gould,^1^ Steven N. Chillrud,^2^ Douglas Phillips,^3^ Matthew S. Perzanowski,^1^ Diana Hernández^3*^

**Affiliations:**

^1^ Department of Environmental Health Science, Mailman School of Public Health of Columbia University, New York, New York, USA

^2^ Lamont-Doherty Columbia Earth Observatory of Columbia University, Palisades, New York, USA

^3^ Department of Sociomedical Sciences, Mailman School of Public Health of Columbia University, New York, New York, USA

**Corresponding Author:** Diana Hernández, dh2494@cumc.columbia.edu, Department of Sociomedical Sciences, 722 W 168^th^ Street, Rm. 546, New York, NY, 10032

**Contents**

1. Methods
   1. Study Recruitment
   2. PM2.5 and BC Measurements
   3. Secondary Analyses
      1. Apartment Characteristics
      2. Behavioral Patterns
      3. I/O Ratios
      4. Meteorological Conditions
2. Results: Supplementary Tables and Figures

**Methods**

**Study Recruitment**

Selected buildings were referenced with official work permits through the New York City Department of Buildings to eliminate buildings that converted their boilers prior to the mandatory conversion date. To recruit apartments, team members attempted to make in-person contact with every apartment on the lower two and upper two floors of the building. For apartments where nobody answered the door, fliers were left with a brief description of the study and contact information. Once two apartments in each building were identified, appointments were scheduled for equipment installation.

Four buildings involved in the study did not convert their boilers to cleaner fuel before the end of the 2014-15 heating season, resulting in the loss of six participants. Six participants did not want to participate again during the post conversion measurements; three apartments in the same buildings of the same floor type were added as replacement. One landlord objected to measurements partway through the pre-conversion measurements, resulting in the loss of two participants. Five participants moved out of their apartments in between the two measurement periods—in four of these five cases occupants of the same apartment or of a different apartment of the same floor type continued in the study.

**Table A. Study recruitment results.**

|  | **Buildings** | **Apartments** | **Upper Floor** | **Lower Floor** |
| --- | --- | --- | --- | --- |
| Target | 30 | 60 | 30 | 30 |
| **Completed** | **28** | **48** | **25** | **23** |
| Lost to follow-up | 5 | 12 | 6 | 6 |

**Table B. Comparison of apartments lost to follow-up to study sample**

|  | | **Lost to follow-up**  **(n=12)** | **Failed to convert**  **(n=6)** | **Completed study**  **(n=48)** |
| --- | --- | --- | --- | --- |
|  | | **Mean / Percent** | **Mean / Percent** | **Mean / Percent** |
| DESCRIPTIVE STATISTICS | | | | |
| *Sex* | |  |  |  |
| Male | | 58% | 50% | 35% |
| Female | | 42% | 50% | 65% |
| *Age (years)* | | 47.08 | 44.17 | 45.77 |
| *Race/Ethnicity* | |  |  |  |
| Non-Hispanic White | | 25% | 17% | 52% |
| Non-Hispanic Black or African-American | | 8% | 0% | 10% |
| Hispanic or Latino | | 67% | 83% | 27% |
| Asian or Pacific Islander | | 0% | 0% | 8% |
| Multiracial | | 0% | 0% | 2% |
| *Education* | |  |  |  |
| Elementary/Primary | | 8% | 0% | 2% |
| High School/Secondary | | 17% | 17% | 15% |
| 2-Year Community College or Vocational | | 33% | 50% | 8% |
| 4-Year College or Greater | | 42% | 33% | 75% |
| *Household Income* | |  |  |  |
| Below $29,999 | | 42% | 50% | 29% |
| $30,000-$49,999 | | 17% | 17% | 10% |
| Above $50,000 | | 42% | 33% | 56% |
| PARTICIPANT BEHAVIORAL PATTERNS | | | | |
| *Does anyone in the home burn candles, incense, or anything like that indoors?* |  | |  |  |
| Yes | 75% | | 50% | 63% |
| No | 25% | | 50% | 37% |
| *Do any members of your household smoke indoors* |  | |  |  |
| Yes | 17% | | 0% | 10% |
| No | 83% | | 100% | 90% |
| *In the last 12 months, how often has second-hand tobacco smoke entered inside your home from somewhere else in or around the building?* |  | |  |  |
| Monthly or more frequently | 42% | | 67% | 42% |
| Never or less frequently than monthly | 58% | | 33% | 52% |
| *Do you ever open windows because your housing unit was too hot?* |  | |  |  |
| Yes | 83% | | 67% | 73% |
| No | 17% | | 33% | 25% |

| PRE-CONVERSION AIR POLLUTION | | | |
| --- | --- | --- | --- |
| *Indoor measures (μg/m^3^)* | **Mean ± SD (n)** | **Mean ± SD (n)** | **Mean ± SD (n)** |
| PM2.5 | 13.4 ± 9.63 (12) | 11.1 ± 8.27 (6) | 12.8 ± 7.93 (48) |
| BC | 1.44 ± 0.71 (12) | 1.26 ± 0.67 (6) | 1.52 ± 0.75 (48) |
| *Outdoor measures (μg/m^3^)* |  |  |  |
| PM2.5 | 8.01 ± 2.05 (12) | 7.10 ± 0.71 (6) | 8.96 ± 3.57 (47) |
| BC | 1.28 ± 0.24 (12) | 1.23 ± 0.12 (6) | 1.33 ± 0.38 (47) |

*PM_2.5_ and BC measurements*

Indoor samples were collected at 1 meter from the floor and 0.75 meters from any wall, with the tubing contained within polypropalene piping to prevent interference from children and pets. Outdoor samples were collected by placing a panel window unit within a casement window that has built into it a pipe with a rain hat that holds the sampling inlet 0.75 meters from the external wall.

*Secondary Analyses*

Apartment characteristics: We hypothesized that upper floor apartments would be less impacted by local emissions sources relative to apartments located on lower floors because of proximity to the street (e.g., traffic patterns, construction). Therefore, upper floor apartments could display different changes after the heating fuel transition because of greater impacts from building and apartment emissions sources. Homes in buildings converting from No. 6 to cleaner fuels (No. 2 and gas) were hypothesized to show a larger improvement than homes in buildings converting from No. 6 to dirtier fuels (No. 4).

Behavioral patterns: Behavioral patterns, like opening windows, may play an important role in personal exposure to air pollution. To account for such patterns, and certain characteristics of apartments, participant responses to apartment behavioral questions were included in regressions and air pollution analyses. These analyses attempted to disentangle relationships between apartment inhabitants and their outdoor environment.

I/O ratios: This parameter is relevant to understanding indoor sources, penetration of ambient sources, and thus personal exposure to PM_2.5_ and BC (Chen and Zhao 2011). Additionally, correlations between changes in indoor and outdoor air pollution were calculated in both our main subsets and in the secondary apartment subsets described above to clarify their relationship.

Meteorological Conditions: Outdoor temperature determines heating demands, which in turn influences the usage of heating oils and potential indoor and outdoor apartment air pollution. To account for these influences, we analyzed air pollution among subsets of apartments with little temperature change between before and after samples. First, we evaluated univariable regressions of wind speed and temperature and outdoor air pollution before and after transition as well as wind speed change and air pollution changes between monitoring periods. After observing results from the regressions, we conducted analysis of air pollution differences before and after fuel transition among apartments with similar wind speeds and temperatures, respectively, before and after transition. Then, we conducted additional subset analyses among apartments with both similar wind speed and temperature before and after fuel transition.

**Results**

**Fig A. Central site weather before and after transition to clean fuel**
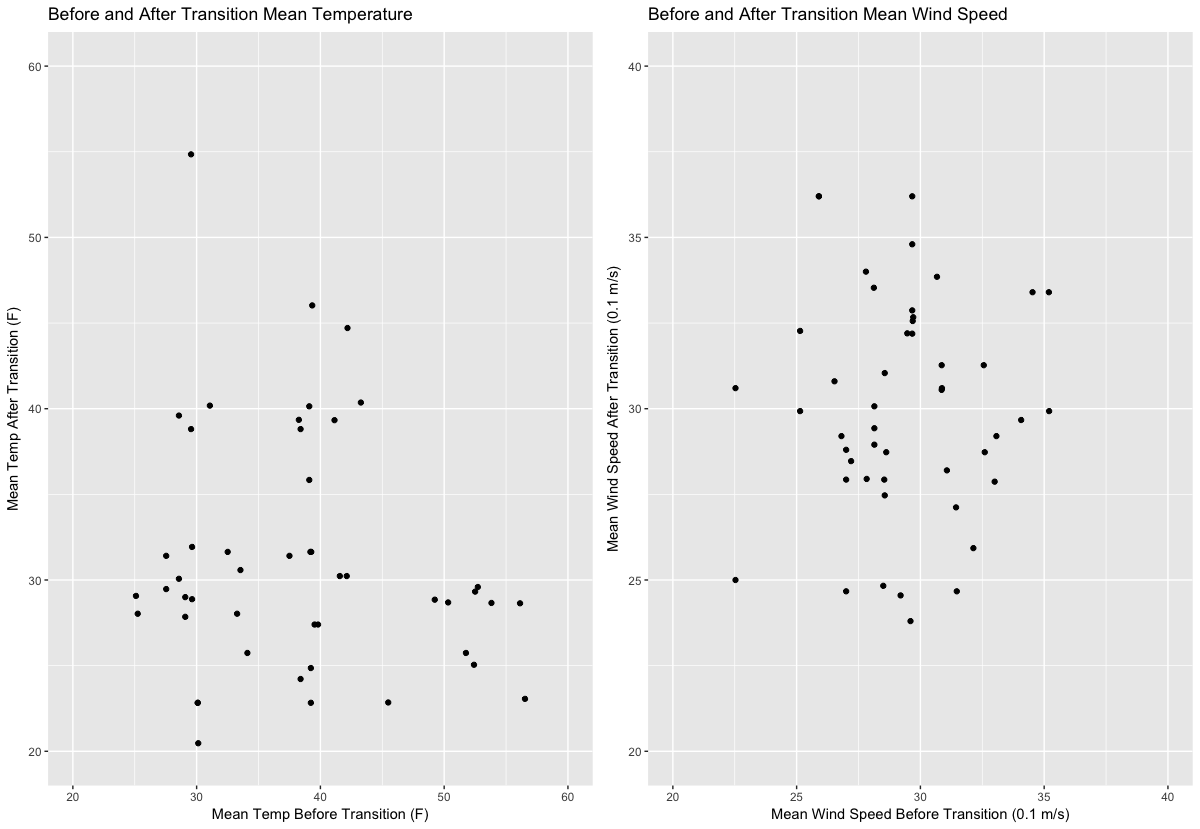


Fig A shows central site weather before and after transition; neither before temperature nor before wind speed is significantly related to their counterparts during the second monitoring period. Given the potential relationship between temperature and wind speed and air pollution, controlling for these differences is important to better understanding observed air pollution measurements.

**Fig B. Central site ambient air quality before and after transition.**
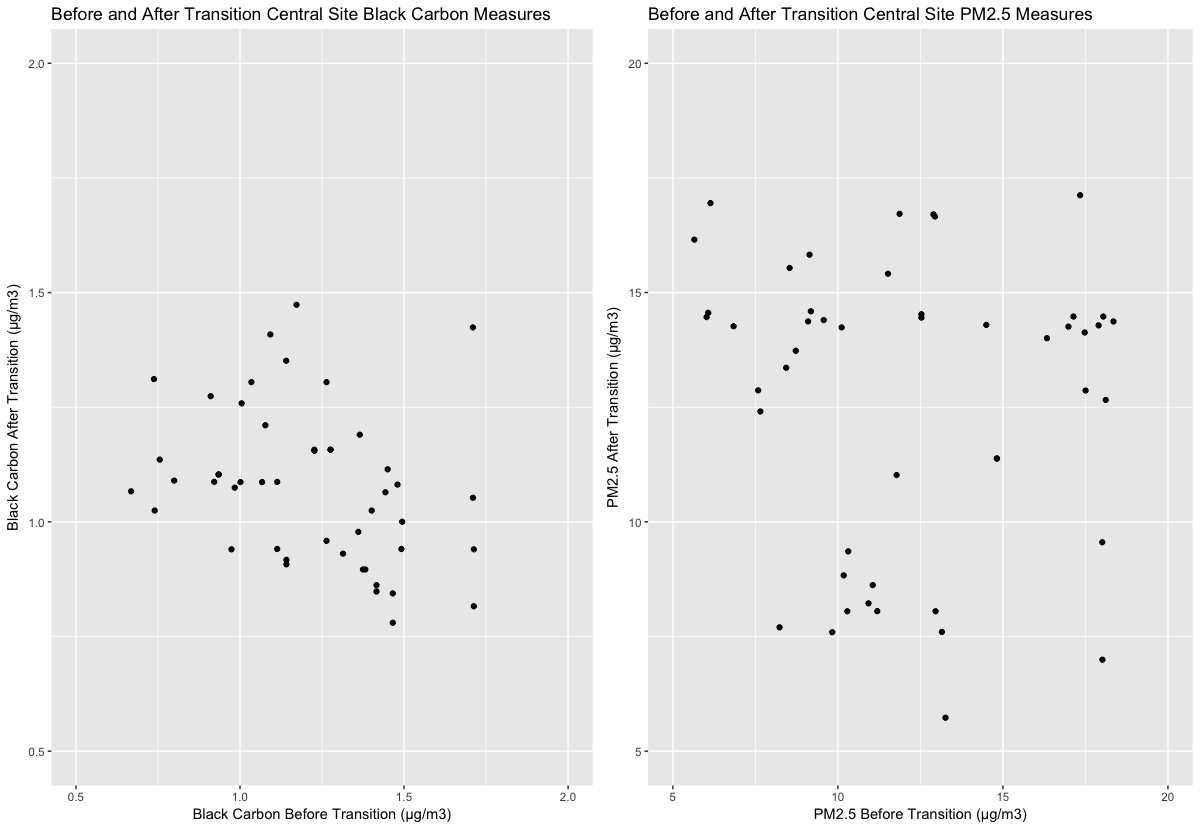


Fig B shows central site ambient air pollution measurements for both BC and PM_2.5_. These results show similar variability between reported measurements during each monitoring period.

**Table C. Descriptive statistics of indoor temperature (^o^F) during sampling**

|  | **Pre-conversion (n=43)** | | **Post-conversion (n=44)** | | **No. 4 oil**  **(n=19)** | | **No. 2 and natural gas**  **(n=25)** | |
| --- | --- | --- | --- | --- | --- | --- | --- | --- |
| *Temperature* |  | *SD* |  | *SD* |  | *SD* |  | *SD* |
| Avg. Temperature | 74.52 | 4.11 | 74.33 | 4.66 | 75.07 | 5.67 | 73.76 | 3.74 |
| Max Temperature | 86.20 | 12.15 | 84.03 | 8.43 | 83.95 | 7.95 | 84.09 | 8.94 |
| Min Temperature | 65.57 | 6.09 | 65.82 | 5.59 | 66.78 | 5.75 | 65.09 | 5.47 |
| Avg. temperature range (Max - Min) | 20.63 | 13.09 | 18.21 | 9.59 | 17.17 | 7.06 | 19.00 | 11.22 |

**Table D. Relationships between observed indoor and outdoor air pollution.**

|  | *Dependent Variable:* | | | | | |
| --- | --- | --- | --- | --- | --- | --- |
|  | Pre Indoor PM2.5 | Pre Indoor BC | | Post Indoor PM2.5 | Post Indoor BC | |
|  | 1 | 2 | 3 | 4 | 5 | 6 |
| Pre Outdoor PM2.5 | -0.121 |  |  |  |  |  |
|  | (0.329) |  |  |  |  |  |
| Pre Outdoor BC |  | 1.066*** |  |  |  |  |
|  |  | (0.227) |  |  |  |  |
| Pre Indoor PM2.5 |  |  | 0.016 |  |  |  |
|  |  |  | (0.014) |  |  |  |
| Post Outdoor PM2.5 |  |  |  | 0.208 |  |  |
|  |  |  |  | (0.875) |  |  |
| Post Outdoor BC |  |  |  |  | 0.493 |  |
|  |  |  |  |  | (0.403) |  |
| Post Indoor PM2.5 |  |  |  |  |  | 0.044*** |
|  |  |  |  |  |  | (0.009) |
| Observations | 47 | 47 | 48 | 48 | 47 | 48 |
| F Statistic | 0.135 (df=1; 46) | 18.451*** (df=1; 46) | 1.284 (df=1; 46) | 0.057 (df=1; 46) | 1.499 (df=1; 46) | 25.057*** (df=1; 46) |
| *Note: *p<0.05; **p<0.01; ***p<0.001* | | | |  |  |  |

Table D compares indoor and outdoor air pollution before and after heating oil transitions in all study apartments. Values shown are regression coefficients for the simple linear relationship between the observed air pollution measurements. Standard errors are in parentheses.

**Table E. Linear regressions of wind speed and observed outdoor air pollution**

|  | *Dependent Variable:* | | | | | |
| --- | --- | --- | --- | --- | --- | --- |
|  | Pre Outdoor BC | Post Outdoor BC | Pre Outdoor PM2.5 | Post Outdoor PM2.5 | Before-After Outdoor BC | Before-After Outdoor PM2.5 |
|  | 1 | 2 | 3 | 4 | 5 | 6 |
| Pre Mean Wind | -0.039* |  | 0.209 |  |  |  |
|  | (0.019) |  | (0.182) |  |  |  |
| Post Mean Wind |  | -0.033* |  | -0.224* |  |  |
|  |  | (0.014) |  | (0.088) |  |  |
| Absolute Wind change | |  |  |  | -0.005 | 0.199 |
|  |  |  |  |  | (0.024) | (0.216) |
| Constant | 2.479*** | 2.280* | 2.862 | 14.848*** | 0.043 | -0.001 |
|  | (0.559) | (0.434) | (5.353) | (2.665) | (0.103) | (0.924) |
| Observations | 47 | 47 | 48 | 47 | 47 | 47 |
| F Statistic  (df=1; 45) | 4.246*) | 5.407* | 1.309 | 6.515* | 0.048 | 0.855 |
| *Note: *p<0.05; **p<0.01; ***p<0.001.* | | | | | | |

Simple linear regressions are shown between pre- and post-mean wind speeds from NOAA Central Park data and measured pre- and post- outdoor air pollution, respectively. Furthermore, simple linear regressions were employed using absolute mean daily wind change between monitoring periods and before-after outdoor air pollution differences (air quality improvements are positive values). Shown are coefficients and standard errors. Constants represent apartments with no daily wind speed or no absolute wind change between monitoring periods. The results indicate a significant relationship between wind speed and air pollution change. These differences are further investigated in subset relationships included in the manuscript main text

**Table F. Air pollutant analysis of apartments with similar meteorological conditions before and after conversion**

|  | **Similar Wind Speed** | | **Similar Temperature** | |
| --- | --- | --- | --- | --- |
|  | *PM2.5* | *BC* | *PM2.5* | *BC* |
| *Indoor measures (μg/m^3^)* | Difference ± SD (n) | Difference ± SD (n) | Difference ± SD (n) | Difference ± SD (n) |
| All | -0.37 ± 14.1 (24) | -0.34 ± 1.17 (24) | -0.08 ± 14.2 (24) | -0.28 ± 1.09 (24) |
| p-value^1^ | 0.9 | 0.17 | 0.98 | 0.23 |
| Clean | 0.50 ± 7.20 (9) | -0.10 ± 0.55 (9) | 0.81 ± 6.28 (12) | -0.15 ± 0.63 (12) |
| p-value | 0.84 | 0.58 | 0.66 | 0.43 |
| Dirty | -0.88 ± 17.2 (15) | -0.48 ± 1.43 (15) | -2.12 ± 20.1 (12) | -0.42 ± 1.39 (12) |
| p-value | 0.85 | 0.68 | 0.73 | 0.35 |
| Upper floor | 2.82 ± 8.09 (12) | -0.36 ± 0.83 (12) | 1.83 ± 9.30 (13) | -0.06 ± 0.52 (12) |
| p-value | 0.48 | 0.16 | 0.49 | 0.67 |
| Lower floor | -3.44 ± 18.2 (13) | -0.32 ± 1.49 (13) | -2.35 ± 18.75 (11) | -0.55 ± 1.46 (11) |
| p-value | 0.51 | 0.48 | 0.69 | 0.24 |
| *Outdoor measures (μg/m^3^)* | *PM2.5* | *BC* | *PM2.5* | *BC* |
| All | 0.82 ± 4.16 (24) | 0.06 ± 0.37 (24) | 1.68 ± 4.11 (23) | 0.06 ± 0.33 (23) |
| p-value^1^ | 0.35 | 0.42 | 0.06 | 0.36 |
| Clean | -1.34 ± 4.39 (9) | 0.01 ± 0.24 (9) | 1.13 ± 2.02 (12) | 0.03 ± 0.39 (12) |
| p-value | 0.39 | 0.89 | 0.08 | 0.83 |
| Dirty | 2.11 ± 3.55 (15) | 0.09 ± 0.44 (15) | 2.28 ± 5.64 (12) | 0.11 ± 0.26 (12) |
| p-value | **0.04** | 0.43 | 0.21 | 0.20 |
| Upper floor | 0.38 ± 3.85 (12) | 0.02 ± 0.35 (12) | 2.42 ± 5.15 (12) | 0.08 ± 0.32 (12) |
| p-value | 0.74 | 0.87 | 0.12 | 0.39 |
| Lower floor | 1.25 ± 4.58 (13) | 0.11 ± 0.40 (13) | 0.71 ± 2.00 (10) | 0.04 ± 0.36 (10) |
| p-value | 0.36 | 0.38 | 0.29 | 0.71 |

Table F shows air pollution differences among apartments with similar meteorological conditions (wind speed and temperature). Clean apartments are those switching to No. 2 or natural gas and dirty apartments are those switching to No. 4 after fuel transition. Improvements in air quality are positive values (before-after differences are shown).

**Table G. Linear Regressions of Temperature Changes and Air Pollution Changes**

|  | *Dependent Variable:* | | | |
| --- | --- | --- | --- | --- |
|  | Before-After Outdoor BC | Before-After Outdoor PM2.5 | Before-After Indoor BC | Before-After Indoor PM2.5 |
|  | 1 | 2 | 3 | 4 |
| Absolute Temperature Change | 0.011* | 0.186** | 0.015 | -0.107 |
|  | (0.006) | (0.094) | (0.017) | (0.173) |
| Constant | -0.016 | -0.053 | -0.314 | 1.109 |
|  | (0.079) | (1.266) | (0.235) | (2.389) |
| Observations | 46 | 46 | 48 | 48 |
| F Statistic | 3.785* (df=1; 44) | 4.040* (df=1; 44) | 0.739 (df=1; 46) | 0.381 (df=1; 46) |
| *Note: *p<0.05; **p<0.01; ***p<0.001* | |  |  |  |

Table G shows simple linear regressions between absolute temperature change between monitoring periods and differences in outdoor and indoor air pollution. Constants represent apartments with no absolute temperature change between monitoring periods. Values shown are regression coefficients and standard errors. Positive values indicate air pollution improvements.

**Table H. Air pollution changes among subsets of apartments with less temperature change**

|  | **Temperature Change (lowest quartile)** | |
| --- | --- | --- |
|  | *PM2.5* | *BC* |
| *Indoor measures (μg/m^3^)* | Difference ± SD (n) | Difference ± SD (n) |
| All | -3.70 ± 18.0 (12) | -0.47 ± 1.45 (12) |
| p-value^1^ | 0.49 | 0.29 |
| Clean | 0.28 ± 8.19 (7) | 0.07 ± 0.75 (7) |
| p-value | 0.93 | 0.82 |
| Dirty | -9.28 ± 27.0 (5) | -1.22 ± 1.94 (5) |
| p-value | 0.48 | 0.2 |
| Upper floor | 1.97 ± 12.1 (8) | -0.06 ± 0.62 (8) |
| p-value | 0.66 | 0.80 |
| Lower floor | -15.0 ± 24.4 (4) | -1.29 ± 2.35 (4) |
| p-value | 0.31 | 0.35 |
| *Outdoor measures (μg/m^3^)^2^* | *PM2.5* | *BC* |
| All | -0.79 ± 2.62 (12) | -0.01 ± 0.37 (12) |
| p-value^1^ | 0.32 | 0.94 |
| Clean | -1.04 ± 2.88 (7) | -0.08 ± 0.18 (7) |
| p-value | 0.38 | 0.30 |
| Dirty | -0.44 ± 2.49 (5) | 0.09 ± 0.56 (5) |
| p-value | 0.72 | 0.73 |
| Upper floor | -0.60 ± 2.18 (8) | -0.11 ± 0.28 (8) |
| p-value | 0.46 | 0.29 |
| Lower floor | -1.16 ± 3.73 (4) | 0.20 ± 0.50 (4) |
| p-value | 0.58 | 0.48 |

Before minus after differences in indoor and outdoor air pollution are shown among apartments with the lowest quartile of temperature change between monitoring periods. In this table, air quality improvements are positive values.

**Table I. Air pollutant analysis by meteorological factors with corrected outdoor measures**

|  | **Similar Wind Speed** | | **Similar Temperature** | |
| --- | --- | --- | --- | --- |
| *Outdoor measures (μg/m^3^)^2^* | *PM2.5* | *BC* | *PM2.5* | *BC* |
| All | 0.82 ± 4.16 (24) | 0.06 ± 0.37 (24) | 1.23 ± 7.04 (23) | -0.01 ± 0.41 (23) |
| p-value^1^ | 0.35 | 0.42 | 0.40 | 0.90 |
| Clean | -1.34 ± 4.39 (9) | 0.01 ± 0.24 (9) | -0.07 ± 2.61 (12) | -0.16 ± 0.22 (12) |
| p-value | 0.39 | 0.89 | 0.92 | **0.03** |
| Dirty | 2.11 ± 3.55 (15) | 0.09 ± 0.44 (15) | 2.66 ± 9.86 (11) | 0.15 ± 0.51 (11) |
| p-value | **0.04** | 0.43 | 0.39 | 0.34 |
| Upper floor | 0.38 ± 3.85 (12) | 0.02 ± 0.35 (12) | 2.41 ± 9.07 (12) | 0.01 ± 0.38 (12) |
| p-value | 0.74 | 0.87 | 0.36 | 0.92 |
| Lower floor | 1.25 ± 4.58 (13) | 0.11 ± 0.40 (13) | -0.30 ± 2.60 (10) | -0.01 ± 0.47 (10) |
| p-value | 0.36 | 0.38 | 0.73 | 0.94 |

Before minus after differences of corrected (adjusted for reference site measures) indoor and outdoor air pollution are shown among apartments with similar wind speed or temperature between monitoring periods. In this table, air quality improvements are positive values.

**Table J. Secondary analysis by meteorologically similar apartments**

|  | *PM2.5* | *BC* |
| --- | --- | --- |
| *Indoor measures (μg/m^3^)* | Difference ± SD (n) | Difference ± SD (n) |
| All | -0.22 ± 19.1 (12) | -0.39 ± 1.44 (12) |
| p-value^1^ | 0.97 | 0.37 |
| Clean | 1.71 ± 1.33 (5) | -0.08 ± 0.71 (5) |
| p-value | **0.04** | 0.81 |
| Dirty | -1.61 ± 25.8 (7) | -0.61 ± 1.82 (7) |
| p-value | 0.87 | 0.41 |
| Upper floor | 5.32 ± 9.61 (6) | -0.08 ± 0.57 (6) |
| p-value | 0.23 | 0.75 |
| Lower floor | -5.77 ± 25.3 (6) | -0.70 ± 2.00 (6) |
| p-value | 0.60 | 0.43 |
| *Outdoor measures (μg/m^3^)^2^* | *PM2.5* | *BC* |
| All | 1.23 ± 1.80 (12) | 0.14 ± 0.23 (12) |
| p-value^1^ | **0.04** | 0.06 |
| Clean | 1.41 ± 2.25 (5) | 0.13 ± 0.29 (9) |
| p-value | 0.23 | 0.36 |
| Dirty | 1.10 ± 1.58 (7) | 0.14 ± 0.19 (15) |
| p-value | 0.12 | 0.11 |
| Upper floor | 1.16 ± 1.43 (6) | 0.06 ± 0.25 (6) |
| p-value | 0.10 | 0.59 |
| Lower floor | 1.30 ± 2.25 (6) | 0.22 ± 0.19 (6) |
| p-value | 0.21 | **0.04** |

Before minus after differences of indoor and outdoor air pollution are shown among apartments with **both** similar wind speed and temperature between monitoring periods. In this table, air quality improvements are positive values. Outdoor air pollution measurements are not corrected.

**Table K. Multivariable linear regressions of household behavioral influences on observed air pollution differences before and after fuel transition**

|  | *Dependent Variable:* | | | |
| --- | --- | --- | --- | --- |
|  | Before-After Outdoor BC | Before-After Outdoor PM2.5 | Before-After Indoor BC | Before-After Indoor PM2.5 |
|  | 1 | 2 | 3 | 4 |
| Opens Windows (=1) | 0.252 | -0.206 |  |  |
|  | (3.479) | (0.367) |  |  |
| Second Hand Smoke Enters (=1) | -8.128* | 0.042 |  |  |
|  | (3.228) | (0.341) |  |  |
| Upper Floor (=1) | 4.064 | -0.187 | 2.329 | 0.080 |
|  | (3.283) | (0.346) | (1.768) | (0.142) |
| Clean Fuel (=1) | 0.486 | -0.052 | -1.956 | -0.065 |
|  | (3.250) | (0.343) | (1.778) | (0.143) |
| Constant | 1.198 | 0.071 | 1.418 | 0.029 |
|  | (4.391) | (0.463) | (1.500) | (0.120) |
| Observations | 46 | 46 | 45 | 45 |
| F Statistic | 1.778* (df=4; 41) | 0.124 (df=4; 41) | 1.503 (df=2; 42) | 0.226 (df=2; 42) |
| *Note: *p<0.05; **p<0.01; ***p<0.001* | |  |  |  |

Multivariable linear regressions of household characteristics and behavioral patterns against differences in outdoor and indoor air pollution. Constants are households that report to not open windows and not have second hand smoke enter the household, and are lower floor and converted to No. 4 fuel oil. Values shown are regression coefficients and standard errors.

**Table L. Secondary analyses of indoor and uncorrected outdoor air pollution changes among apartment subsets reporting to open windows**

|  | **Opens Windows** | |
| --- | --- | --- |
|  | *PM2.5* | *BC* |
| *Indoor measures (μg/m^3^)* | Difference ± SD (n) | Difference ± SD (n) |
| All | 0.19 ± 10.9 (33) | -0.27 ± 1.22 (33) |
| p-value^1^ | 0.92 | 0.22 |
| Clean | 2.17 ± 3.71 (13) | -0.27 ± 0.45 (13) |
| p-value | 0.06 | 0.05 |
| Dirty | -1.09 ± 13.7 (20) | -0.26 ± 1.55 (20) |
| p-value | 0.73 | 0.45 |
| Upper floor | 0.06 ± 4.91 (15) | -0.43 ± 0.66 (15) |
| p-value | 0.96 | **0.02** |
| Lower floor | 0.30 ± 14.3 (18) | -0.13 ± 1.56 (18) |
| p-value | 0.93 | 0.72 |
| *Outdoor measures (μg/m^3^)^2^* | *PM2.5* | *BC* |
| All | 3.14 ± 6.42 (33) | 0.02 ± 0.50 (33) |
| p-value^1^ | **0.01** | 0.76 |
| Clean | 2.10 ± 4.06 (13) | 0.05 ± 0.34 (13) |
| p-value | 0.09 | 0.61 |
| Dirty | 3.85 ± 7.66 (20) | 0.01 ± 0.59 (20) |
| p-value | **0.04** | 0.93 |
| Upper floor | 4.62 ± 8.09 (15) | 0.19 ± 0.36 (15) |
| p-value | **0.04** | 0.07 |
| Lower floor | 1.84 ± 4.32 (18) | -0.11 ± 0.58 (18) |
| p-value | 0.10 | 0.42 |

Table L shows indoor and corrected outdoor air pollution changes among apartments reporting to open windows. Differences are before-after, meaning that positive values indicate improvements in air pollution. Two apartments reporting opening windows are not included because they did not have paired measurements pre- and post-conversion.

**Table M. Analysis by reported presence of second-hand smoke in the apartment**

|  | **Has had second-hand smoke enter home** | | **Has had no second-hand smoke enter home** | |
| --- | --- | --- | --- | --- |
|  | *PM2.5* | *BC* | *PM2.5* | *BC* |
| *Indoor measures (μg/m^3^)* | Difference ± SD (n) | Difference ± SD (n) | Difference ± SD (n) | Difference ± SD (n) |
| All | -4.11 ± 12.4 (20) | -0.18 ± 1.43 (20) | 3.34 ± 8.81 (26) | -0.19 ± 0.74 (26) |
| p-value^1^ | 0.15 | 0.59 | 0.06 | 0.20 |
| Clean | -1.18 ± 7.88 (8) | -0.32 ± 0.26 (9) | 1.83 ± 6.31 (12) | -0.11 ± 0.65 (12) |
| p-value | 0.69 | **0.01** | 0.34 | 0.57 |
| Dirty | -6.06 ± 14.6 (12) | -0.08 ± 1.86 (12) | 4.62 ± 10.6 (14) | -0.26 ± 0.83 (14) |
| p-value | 0.18 | 0.88 | 0.13 | 0.26 |
| Upper floor | 0.51 ± 4.53 (12) | -0.18 ± 0.40 (12) | 2.36 ± 10.5 (11) | -0.33 ± 0.89 (11) |
| p-value | 0.70 | 0.15 | 0.47 | 0.25 |
| Lower floor | -11.04± 17.1 (8) | -0.16 ± 2.30 (8) | 4.05 ± 7.65 (15) | -0.09 ±0.63 (15) |
| p-value | 0.11 | 0.85 | 0.06 | 0.58 |
| *Outdoor measures (μg/m^3^)^2^* | *PM2.5* | *BC* | *PM2.5* | *BC* |
| All | 3.08 ± 7.92 (20) | 0.11 ± 0.33 (20) | 0.76 ± 3.96 (26) | -0.01 ± 0.55 (26) |
| p-value^1^ | 0.11 | 0.16 | 0.34 | 0.91 |
| Clean | 0.78 ± 4.81 (8) | 0.09 ± 0.31 (8) | 0.53 ± 3.92 (12) | -0.06 ± 0.30 (12) |
| p-value | 0.66 | 0.42 | 0.65 | 0.52 |
| Dirty | 4.76 ± 9.45 (12) | 0.12 ± 0.39 (12) | 0.95 ± 4.13 (14) | 0.03 ± 0.71 (14) |
| p-value | 0.13 | 0.27 | 0.40 | 0.89 |
| Upper floor | 3.83 ± 9.20 (12) | 0.04 ± 0.23 (12) | 1.88 ± 3.53 (11) | 0.12 ± 0.46 (11) |
| p-value | 0.18 | 0.53 | 0.11 | 0.40 |
| Lower floor | 1.80 ± 5.49 (8) | 0.23 ± 0.45 (8) | -0.07 ± 4.17 (15) | -0.11 ± 0.60 (15) |
| p-value | 0.43 | 0.22 | 0.95 | 0.49 |

Table M shows indoor and (uncorrected) outdoor air pollution changes among apartments reporting to have had second-hand smoke enter their home and those not reporting having had second-hand smoke enter their home. Differences are before-after, meaning that positive values indicate improvements in air pollution.

**Table N. Associations between building characteristics and indoor air pollution**

|  | **Before PM2.5 Indoor** | **After PM2.5 Indoor** | **PM2.5 Difference** | **Before BC Indoor** | **After BC Indoor** | **BC Difference** |
| --- | --- | --- | --- | --- | --- | --- |
| *Building Age (Years)* | -0.28 (0.03)* | -0.08 (0.70) | -0.15 (0.40) | 0.01 (0.49) | -0.01 (0.38) | 0.02 (0.22) |
| *Boiler Capacity*  *(Gross BTU)* | 0.18 (0.81) | -2.65 (0.02)* | 3.21 (0.001)** | -0.04 (0.6) | -0.11 (0.21) | 0.08 (0.44) |
| *Boiler Age Range:* |  |  |  |  |  |  |
| <10 Yrs. | 5.27 (0.27) | 9.30 (0.17) | -3.82 (0.52) | -0.76 (0.08) | 0.46 (0.36) | -1.21 (0.05) |
| 11-20 Yrs. | 3.48 (0.44) | 3.46 (0.60) | -1.18 (0.84) | -0.46 (0.25) | 0.22 (0.64) | -0.6 (0.31) |
| 21-30 Yrs. | 5.62 (0.25) | -0.23 (0.97) | 6.89 (0.27) | -0.69 (0.12) | -0.16 (0.76) | -0.58 (0.36) |
| >30 Yrs. | Ref | Ref | Ref | Ref | Ref | Ref |

Table N shows results from simple linear regressions between building characteristics and apartments’ before, after, and differences in before minus after indoor air pollution. Coefficients and p-values (in parentheses) shown are shown.

**Table O. Multivariable linear regression of building characteristics and indoor air pollution**

|  | **Before PM2.5 Indoor** | | **After PM2.5 Indoor** | | **PM2.5 Difference** | | **Before BC Indoor** | | **After BC Indoor** | | **BC Difference** | |
| --- | --- | --- | --- | --- | --- | --- | --- | --- | --- | --- | --- | --- |
| *Building Age (Years)* | -0.32 | (0.03)* | -0.15 | (0.51) | -0.09 | (0.60) | 0.01 | (0.69) | -0.01 | (0.40) | 0.02 | (0.31) |
| *Boiler Capacity*  *(Gross BTU)* | 6.40 | (0.22) | 3.00 | (0.70) | 4.99 | (0.42) | -1.10 | (0.02)* | 0.19 | (0.74) | -1.30 | (0.07) |
| *Boiler Age:* |  |  |  |  |  |  |  |  |  |  |  |  |
| <10 Years | 8.43 | (0.09) | -1.61 | (0.82) | 10.99 | (0.06) | -0.88 | (0.05) | -0.16 | (0.77) | -0.74 | (0.26) |
| 11-20 Years | 4.90 | (0.28) | 2.26 | (0.73) | 1.31 | (0.80) | -0.60 | (0.14) | 0.20 | (0.69) | -0.68 | (0.26) |
| 21-30 Years | 0.14 | (0.88) | -2.55 | (0.09) | 3.51 | (0.01)* | -0.12 | (0.15) | -0.11 | (0.32) | -0.01 | (0.94) |
| >30 Years | Ref |  | Ref |  | Ref |  | Ref |  | Ref |  | Ref |  |
| *Clean Heating Fuel (=1)* | 0.61 | (0.79) | -1.36 | (0.71) | 0.84 | (0.77) | -0.20 | (0.34) | -0.06 | (0.83) | -0.22 | (0.51) |

Table O shows results from multivariable linear regressions between building characteristics and apartments’ before, after, and differences in before minus after indoor air pollution. Coefficients and p-values (in parentheses) shown are shown. All variables are included in regressions.

**Table P. Indoor/outdoor ratios for measured pollutants**

|  | **All Households**  **(#6 to any fuel)** | **#6 to #2 or gas (cleaner fuels)** | **#6 to #4 (dirtier fuel)** |
| --- | --- | --- | --- |
| *BC* | *Mean ± SD (n)* | *Mean ± SD (n)* | *Mean ± SD (n)* |
| Pre-conversion | 1.16 ± 0.50 (46) | 1.00 ± 0.15 (21) | 1.30 ± 0.64 (25) |
| Post conversion | 1.39 ± 0.76 (46) | 1.27 ± 0.43 (21) | 1.49 ± 0.96 (25) |
| Upper floor |  |  |  |
| Pre-conversion | 1.02 ± 0.16 (24) | 0.94 ± 0.12 (11) | 1.08 ± 0.17 (13) |
| Post conversion | 1.32 ± 0.66 (24) | 1.17 ± 0.42 (11) | 1.45 ± 0.80 (13) |
| Lower floor |  |  |  |
| Pre-conversion | 1.32 ± 0.67 (22) | 1.06 ± 0.15 (10) | 1.54 ± 0.85 (12) |
| Post conversion | 1.46 ± 0.87 (22) | 1.38 ± 0.44 (10) | 1.53 ± 1.16 (12) |
| *PM2.5* |  |  |  |
| Pre-conversion | 1.55 ± 1.04 (46) | 1.44 ± 0.92 (21) | 1.65 ± 1.15 (25) |
| Post conversion | 1.67 ± 1.84 (46) | 1.72 ± 2.27 (21) | 1.62 ± 1.42 (25) |
| Upper floor |  |  |  |
| Pre-conversion | 1.57 ± 1.05 (24) | 1.65 ± 1.18 (11) | 1.51 ± 0.96 (13) |
| Post conversion | 1.68 ± 2.10 (24) | 2.10 ± 3.03 (11) | 1.33 ± 0.70 (13) |
| Lower floor |  |  |  |
| Pre-conversion | 1.55 ± 1.07 (22) | 1.22 ± 0.45 (10) | 1.82 ± 1.35 (12) |
| Post conversion | 1.65 ± 1.56 (22) | 1.30 ± 0.96 (10) | 1.96 ± 1.95 (12) |

Table P shows pre- and post-conversion indoor/outdoor ratios as divided among all apartments and among those transitioning to cleaner fuels (No. 2 or natural gas) and among those transitioning to comparatively dirtier No. 4 oil. Values over 1 indicate that air pollution measurements are higher indoors as compared to outdoors. Two apartment measurements have been dropped because of lack of an outdoor measurement pre- and post-conversion.
