# Supplementary material for: Soot and the city: Evaluating the impacts of Clean Heat policies on indoor/outdoor air quality in New York City apartments
Source: PLoS One. 2018 Jun 28;13(6):e0199783. doi: 10.1371/journal.pone.0199783 (PMC6023219; doi:10.1371/journal.pone.0199783)
Supplement: S2 File — (DOCX) [file pone.0199783.s002.docx]

Survey Instrument

Participant Code Number: __________________

Interviewer Name: __________________

Interview Date: ____________________

PC1. Pre or Post conversion measurements

❑ Pre………………...1

❑ Post ……………....2

SVY. Did participant complete Survey?

❑ Yes………………...1

❑ No ………………....0

P1. Same or different participants and apartments between pre and post conversion measurements?

❑ Same participant and same apartment pre-conversion measurements completed year one, post conversion measurements completed year two…..…1

❑ Same participant and same apartment pre and post conversion measurements completed in year two……………………………………………….2

❑ Different participant, different apartment pre-conversion measurements completed year one, post conversion measurements completed year two….......3

❑ Different participant, different apartment pre and post conversion measurements completed in year two ………………………………………………4

❑ Same apartment, different participant pre-conversion measurements completed year one, post conversion measurements completed year two……...5

❑ Same apartment, different participant pre and post conversion measurements completed in year two ………………………………………………………..……..…6

❑ Pre-conversion data year one only…………………………………….……….7

❑ Pre conversion data year two only……………………………………...….…...8

❑ Did not complete survey ………………………………………….....….…...99999

F1. Upper or Lower floor apartment?

❑ Lower floor ……………..…....1

❑ Upper floor……………..…….2

❑ Did not complete survey …...99999

BL. Building Number

❑ Building 1............................1

❑ Building 2………………...…2

❑ Building 3.………….....…....3

❑ Building 4…………..………4

❑ Building 5……………......…5

❑ Building 6…………….….…6

❑ Building 7…………….….…7

❑ Building 8………………..…8

❑ Building 9……………......…9

❑ Building 10…….………...…10

❑ Building 11……………....…11

❑ Building 12……………....…12

❑ Building 13………….…...…13

❑ Building 14……………....…14

❑ Building 15……………....…15

❑ Building 16……………....…16

❑ Building 17……………....…17

❑ Building 18...........................18

❑ Building 19…………..…..…19

❑ Building 20.………….......…20

❑ Building 21…………..…..…21

❑ Building 22……………....…22

❑ Building 23…………………23

❑ Building 24…………………24

❑ Building 25…………………25

❑ Building 26……………....…26

❑ Building 27…….………...…27

❑ Building 28……………....…28

❑ Building 29……………....…29

❑ Building 30………….…...…30

❑ Building 31………….…...…31

❑ Building 32………….…...…32

❑ Building 33………….…...…33

❑ Did not complete survey ....99999

FT. Fuel Type in Building

❑ #6 Oil..................................1

❑ #4 Oil………………..…..…2

❑ #2 Oil.…………...........…....3

❑ Natural Gas………..........…4

❑ Did not complete survey ...99999

**Household Demographics:**

HD2. Sex of participant? 1…Male 0…Female

❑ Did not complete survey .….…...99999

HD3. What is your age? _____

❑ Did not complete survey …...99999

HD4. What is the highest level of education you have completed?

❑ Elementary/Primary School ……………….............................…1

❑ High School/Secondary School Diploma or equivalent……….2

❑ 4 Year College Degree…………………………………………....4

❑ 2 Year Community College Degree………….............................5

❑ Vocational School ………………………...…………………….…6

❑ Post-Graduate Degree……………………..................................7

❑ Did not complete survey ……………………………………….....99999

HD5. Please specify your race or ethnicity. (*Check all that apply*)

❑ Non-Hispanic White …………………………1

❑ Non- Hispanic Black or African American…2

❑ Hispanic or Latino ……………………………3

❑ Asian or Pacific Islander……………………..5

❑ Multiracial…………………………….……..…7

❑ Did not complete survey ……………...……...99999

HD6. Are you currently…

❑ Employed for wages ………………………………1

❑ Self-employed……………………………………...2

❑ Out of work but looking for work …………………3

❑ Out of work but not currently looking for work…..4

❑ A student……………………………………………6

❑ Retired ……………………………………………...7

❑ Unable to work (disability)…………………….…..8

❑ Did not complete survey …………………..……...99999

HD7. What is your annual household income?

❑ Less than $10,000 …………..1

❑ $10,000-$19,999……………..2

❑ $20,000-$29,999 ………….…3

❑ $30,000-$39,999………….….4

❑ $40,000-$49,999………….….5

❑ $50,000 or more……………...6

❑ Did Not answer…………….…9

❑ Did not complete survey ….....99999

HD8. What are your sources of income? Do you receive…

❑ Salary ………………………………………........1

❑ Pension…………………………………………..2

❑ Social Security ………………………………….3

❑ TANF…………………………………………..….4

❑ Investments……………………………………....5

❑ Child Support………………………………….…6

❑ Scholarships.………………………………….…7

❑ Student loans………………………………….…8

❑ No response……………………………………...9

❑ Money from family………………………………10

❑ Salary & Pension……………………….………12

❑ Salary & social security………………………...13

❑ Social security and investments………………15

❑ Pension & social security………………………23

❑ Social security & investments…………………35

❑ Salary, pension, & investments………….....…125

❑ Pension, social security, & investments …..…235

❑ Did not complete survey ………..….....….…....99999

HD9. The place where you live, do you… [CHOOSE ONLY ONE]

❑ Own…………………………...…...1

❑ Rent……………………...…….......2

❑ Did not complete survey ……...99999

HD10. Do you split rent with a roommate or other housemate?

❑ Yes……………………………...1

❑ No ……………………………..0

❑ Was not asked .......................9

❑ Did not complete survey …...99999

HD10a. How much money per month do you pay in rent?

$______________________

❑ Did not answer ...................................99

❑ Not applicable (owns doesn’t rent) ....88

❑ Was not asked…….. ..........................77

❑ Did not complete survey …………...99999

HD11. Are you receiving any sort of housing benefits?

❑ Yes………………...……….….....1

❑ No [SKIP TO HD12]..…………...0

❑ Did not complete survey ….…...99999

HD11a. What kind of benefits are you receiving?

❑ Section 8 building ………………....………3

❑ Rent Controlled ... …………………………4

❑ Rent Subsidized... …………………………5

❑ Senior citizen rent increase waiver............6

❑ Not applicable………….…………………...9

❑ Did not complete survey ……………...…...99999

HD12. How many people live in your household, including yourself?

Number of people in household: ______

❑ Did not complete survey ………...99999

IF HD12 IS “1”, SKIP TO S1

IF H12 IS MORE THAN “1”:

HD12a. Who are the other people who live with you? (*Check all that apply*)

❑ Spouse………………………………………….1

❑ Unmarried partner …………………………….2

❑ Child…………………………………………….3

❑ Grandchild……………………………………...4

❑ Parent (Mother/Father) ……………………….5

❑ Brother/Sister………………………….………..6

❑ Other family member…....................................7

❑ Foster child……………………………….……..8

❑ Housemate/Roommate ...................................9

❑ Roomer/Boarder…………………………….…10

❑ Other non-relative……………………………..11

❑ Spouse and child(ren)……………………..….13

❑ Unmarried partner & child(ren)……………….23

❑ Child(ren) & grandchild(ren)…………….…….34

❑ Child(ren) & other family member…………….37

❑ Spouse, unmarried partner, & child(ren)…….123

❑ Spouse, child(ren), & parent……………….….135

❑ Not applicable………………………………...…99

❑ Did not complete survey ……………....……...99999

HD12b. What are the ages of people living in your household under the age of 18?

❑ 1 year .................................1

❑ 2 years …………..…………2

❑ 3 years .…………............…3

❑ 4 years …………..…………4

❑ 5 years ………………......…5

❑ 6 years …………………..…6

❑ 7 years ……………….….…7

❑ 8 years …………………..…8

❑ 9 years ……………..........…9

❑ 10 years …….…………..…10

❑ 11 years ………………...…11

❑ 12 years ………………...…12

❑ 13 years ………….……..…13

❑ 14 years ………………...…14

❑ 15 years ………………...…15

❑ 16 years ………………....…16

❑ 17 years ………………....…17

❑ 4 & 1 years……………....…41

❑ 4 & 2 years………….…...…42

❑ 49 years………………….…49

❑ Under 1 year old……………100

❑ 1 & 1 year…………...........…101

❑ 1 & 11 years……..............…111

❑ 1 & 15 years…………….......115

❑ 8 & 13 years…………….......813

❑ 11 & 11 years…………..…...1111

❑ 11 & 14 years…….…….…...1114

❑ 12 & 12 years………….……1212

❑ 12 & 14 years…………….…1214

❑ 17 & 14 years…………….....1714

❑ 2, 7, & 13 years………….….2713

❑ 12, 14, & 3 years…………....12143

❑ 11, 13, & 15 years………..…111315

❑ Nobody under 18 in home…99

❑ Did not complete survey…....99999

S1. How long have you lived in this apartment?

S1 Months.________

S1 Years._______

❑ Was not asked ..........................88

❑ Did not answer ..........................99

❑ Did not complete survey ….......99999

S2. Have you ever had any challenges with your housing situation, such as concerns about not being able to afford rent or homelessness?

❑ Yes……………….………….....1

❑ No [SKIP TO HS1] …………...0

❑ Don’t Know…………………....8

❑ Was not asked ........................99

❑ Did not complete survey ….....99999

S3. In the last year have you been homeless at any time? That is, have you ever slept outside, in a car or in a homeless shelter?

❑ Yes………………………..…..1

❑ No ………………………….....0

❑ Don’t Know…………………...8

❑ Not applicable………………..9

❑ Did not complete survey …....99999

S4. In the last year, has there been a time when you couldn’t afford a place to stay, or when you couldn’t pay the rent?

❑ Yes…………………………....1

❑ No ………………………….....0

❑ Don’t Know………………......8

❑ Not applicable…………….....9

❑ Did not complete survey …...99999

S5. In the last year, has there been a time when you or a member of your family needed medicine or medical care but didn't get the treatment because you couldn't afford it?

❑ Yes…………………………..1

❑ No …………………………...0

❑ Don’t Know………………....8

❑ Not applicable……………...9

❑ Did not complete survey …..99999

**Health Survey (SF-12):**

HS1. During the winter months, during the week, meaning Monday through Friday, how much time do you spend inside your home during the day? Would you say you spend…

❑ All day inside…………….…4

❑ Most of the day inside……..3

❑ Some of the day inside……2

❑ A little of the day inside……1

❑ None of the day inside….…0

❑ Don’t know……………….…8

❑ Did not complete survey......99999

HS1a. Does this change depending on the season? (Probe: for example, when it is warmer outside in the summer do you spend more or less time inside?)

❑ Yes out more in summer....1

❑ No ………………….…….....0

❑ Don’t Know………………...8

❑ No response……………….9

❑ Did not complete survey.....99999

HS2. During the winter, during the weekend, meaning Saturday and Sunday, how much time do you spend inside your home during the day? Would you say you spend…

❑ All day inside…………….…4

❑ Most of the day inside……..3

❑ Some of the day inside……2

❑ A little of the day inside……1

❑ None of the day inside….…0

❑ Don’t know……………….…8

❑ Did not complete survey …..99999

HS2a. Does this change depending on the season? (Probe: for example, when it is warmer outside in the summer do you spend more or less time inside?)

❑ Yes out more in summer…...1

❑ No ……………………….…...0

❑ Don’t Know…..……………....8

❑ No response…………….…...9

❑ Did not complete survey …...99999

HS3. In general, would you say your health is:

❑ Excellent………………………5

❑ Very good……………………..4

❑ Good ………………………….3

❑ Fair ………………………….…2

❑ Poor……………………………1

❑ Don’t know……………………8

❑ Did not complete survey …....99999

HS4. The following items are about activities you might do during a typical day. Does your health now limit you in these activities? If so, how much?

HS4. Moderate activities, such as moving a table, pushing a vacuum cleaner, bowling, or playing golf?

❑ Yes, limited a lot………………………1

❑ Yes, limited a moderate amount…….2

❑ Yes, limited a little. ………………...…3

❑ No, not limited at all………………......0

❑ Don’t Know………..………………......8

❑ Did not complete survey ……….........99999

HS4a. Climbing several flights of stairs.

❑ Yes, limited a lot………………………1

❑ Yes, limited a moderate amount…….2

❑ Yes, limited a little. ………………...…3

❑ No, not limited at all………………......0

❑ Don’t Know………..………………......8

❑ Did not complete survey..……….…...99999

HS5. During the past 4 weeks, have you had any of the following problems with your work or other regular daily activities as a result of your physical health?

HS5. Accomplished less than you would like?

❑ Yes……………………….…....1

❑ No ……………………….….....0

❑ Don’t Know……………….......8

❑ Did not complete survey …....99999

HS5a. Were limited in the kind of work or other activities

❑ Yes………………………….....1

❑ No …………………………......0

❑ Don’t Know…………………...8

❑ Did not complete survey …....99999

HS6. During the past 4 weeks, have you had any of the following problems with your work or other regular daily activities as a result of any emotional problems (such as feeling depressed or anxious)?

HS6. Accomplished less than you would like?

❑ Yes………………………….....1

❑ No …………………………......0

❑ Don’t Know…………………...8

❑ Did not complete survey …....99999

HS6a. Didn't do work or other activities as carefully as usual?

❑ Yes………………………....1

❑ No …………………….…....0

❑ Don’t Know………………..8

❑ Did not complete survey....99999

HS7. During the past 4 weeks, how much did pain interfere with your normal work (including both work outside the home and housework)?

❑ Not at all ………………..…0

❑ Slightly………………..……1

❑ Moderately ………………..2

❑ Quite a bit ……………..…..3

❑ Extremely………………..…4

❑ Don’t Know………………...8

❑ Did not complete survey ....99999

HS8. These questions are about how you feel and how things have been with you during the past 4 weeks. For each question, please give the one answer that comes closest to the way you have been feeling. How much of the time during the past 4 weeks…

HS8. have you felt calm and peaceful?

❑ All of the time………………...5

❑ Most of the time …………..…4

❑ A good bit of the time …….....3

❑ Some of the time …………....2

❑ A little of the time ………..…..1

❑ None of the time…………...…0

❑ Don’t Know……………...…....8

❑ Did not complete survey …....99999

HS8a. did you have a lot of energy?

❑ All of the time…………...…..5

❑ Most of the time ………....…4

❑ A good bit of the time …......3

❑ Some of the time …………..2

❑ A little of the time …………..1

❑ None of the time………….…0

❑ Don’t Know……………….....8

❑ Did not complete survey …..99999

HS8b. have you felt downhearted and blue?

❑ All of the time………………...5

❑ Most of the time ………….…4

❑ A good bit of the time ……....3

❑ Some of the time …………...2

❑ A little of the time ……….…..1

❑ None of the time………….…0

❑ Don’t Know………………......8

❑ Did not complete survey …...99999

HS9. During the past 4 weeks, how much of the time has your physical health or emotional problems interfered with your social activities (like visiting friends, relatives, etc.)?

❑ All of the time………………...4

❑ Most of the time …………..…3

❑ Some of the time ………..…..2

❑ A little of the time ……….…...1

❑ None of the time…………..…0

❑ Don’t Know…………………...8

❑ Did not complete survey …....99999

BC1. Does anyone in the home burn candles, incense, or anything like that indoors?

❑ Yes………………………….….1

❑ No [SKIP TO T1]……………...0

❑ Did not complete survey ….....99999

BC1A. How often would you say those things are burned indoors? Would you say

❑ More than once a day……………………….5

❑ Once a day…………………………………...4

❑ Between once a day and once a week……3

❑ Once a week…………………………………2

❑ Less than once a week……………………..1

❑ Don’t Know…………………………………..8

❑ Not applicable…………………………….…99

❑ Did not complete survey …………….…......99999

BC1B. Where are those things burned in the apartment?

❑ Living Room……………………………...…….1

❑ Dining Room………….………………..….…...2

❑ Bedroom (Adult)………..……………….…..…3

❑ Bedroom (child under 18)…………..………...4

❑ Kitchen……………………………….…..…….5

❑ Bathroom……………………………….………6

❑ Study……………………………………....……7

❑ Entrance………………………….……..………8

❑ hallway……………………………………..……9

❑ Foyer…………………………………….………10

❑ Living room & dining room………………...…12

❑ Living room & bedroom (adult)………………13

❑ Living room & kitchen…………………………15

❑ Bedroom (adult) & kitchen……………………35

❑ Living room, Bedroom (adult), & kitchen……135

❑ Living room, bedroom (adult) & hallway….…139

❑ Living room, kitchen, & bathroom……………156

❑ Dining room, bathroom, & hallway………..…269

❑ All rooms in the home………………………...100

❑ Was not Asked…………………………….…...88

❑ Not applicable…………………………….……99

❑ Did not complete survey ………………….…..99999

T1. Do any members of your household smoke indoors?

❑ Yes, cigarettes……………..…1

❑ Yes, marijuana………………..2

❑ No [SKIP TO T2]…………..….0

❑ Did not complete survey ….....99999

T2. Do any visitors to your home smoke indoors?

❑ Yes………………………….….1

❑ No ……………………………...0

❑ Did not complete survey ….....99999

T3. In the last 12 months, how often has second-hand tobacco smoke entered inside your home from somewhere else in or around the building? Would you say it was…

❑ Daily…………………….…….1

❑ Weekly………………….…….2

❑ Monthly………………….……3

❑ A few times……………….…..4

❑ Never……………………….…0

❑ Don’t Know……………….…..8

❑ Did not complete survey …....99999

**Asthma and Health Questions**

AS1. (From Phone Survey, Appendix C) Have you or anyone in your household ever been told by a doctor nurse or other health professional that they have any of the following?

FOR AS1a-AS1L……❑ Did not complete survey …...99999

| AS1a. | Asthma | ❑ Yes…1 | ❑ No…0 | ❑ Dk…8 |
| --- | --- | --- | --- | --- |
| AS1b. | Chronic respiratory, lung or breathing trouble (NOT ASTHMA) | ❑ Yes…1 | ❑ No…0 | ❑ Dk…8 |
| AS1c. | Chronic allergies or sinus trouble | ❑ Yes…1 | ❑ No…0 | ❑ Dk…8 |
| AS1d. | Chronic orthopedic, bone or joint problems | ❑ Yes…1 | ❑ No…0 | ❑ Dk…8 |
| AS1e. | Depression | ❑ Yes…1 | ❑ No…0 | ❑ Dk…8 |
| AS1f. | Developmental Delay or cognitive impairment | ❑ Yes…1 | ❑ No…0 | ❑ Dk…8 |
| AS1g. | Hypertension | ❑ Yes…1 | ❑ No…0 | ❑ Dk…8 |
| AS1h. | Epilepsy (seizure disorder) | ❑ Yes…1 | ❑ No…0 | ❑ Dk…8 |
| AS1i. | Learning problems | ❑ Yes…1 | ❑ No…0 | ❑ Dk…8 |
| AS1j. | Heart Disease | ❑ Yes…1 | ❑ No…0 | ❑ Dk…8 |
| AS1k. | Neurological Disorders | ❑ Yes…1 | ❑ No…0 | ❑ Dk…8 |
| AS1L. | Stroke | ❑ Yes…1 | ❑ No…0 | ❑ Dk…8 |

AS1m. Any other recurring illnesses not mentioned here?

❑ Eczema ………………………1

❑ High blood pressure...........…2

❑ Diabetes ……………..........…3

❑ Too much iron …………….…4

❑ Gastritis …………………........5

❑ Hyperlipidemia …………....…6

❑ Arthritis ………………….....…7

❑ Breast cancer ……………..…8

❑ Diabetes …………………...…9

❑ Viral infection ……………..…10

❑ Migraines ………………….…11

❑ Fibromyalgia ………...........…12

❑ Did not answer…………....…98

❑ Not applicable…………….…99

❑ Did not complete survey …...99999

AS2. Is anyone in your household **currently** under the care of a doctor, medical provider or clinic for any health problems?

❑ Yes ……………………………….1

❑ No [SKIP TO AS3]………….......0

❑ Don’t Know [SKIP TO AS3] ...…8

❑ No response [SKIP TO AS3]…...9

❑ Did not complete survey …...…...99999

AS2a. Could you please explain the health problem that they are under care for?

❑ Migraines …………………………………..…....……1

❑ Depression...........................................................…2

❑ Eczema ………………………………………….....…3

❑ Scoliosis & eczema ……………………………….…4

❑ HIV ……………………………………….………....…5

❑ Psoriasis …………………………………………...…6

❑ Asthma & shoulder problem ………….…….….....…7

❑ Diabetes ………………………………………………8

❑ Hypertension……………………………….……....…9

❑ Heart Condition …………………………………..…10

❑ Respiratory condition………………………….……11

❑ Minor cut.............................................................…12

❑ Chest pain………………………………………...…13

❑ Infection …………………………………………..…14

❑ Allergic reaction ……………...............................…15

❑ Sinus & respiratory issues……………………....…16

❑ Recovery from surgery ………………………......…17

❑ Psychological therapy …………………………...…18

❑ Back injury ………………………………………...…19

❑ Hyperlipidemia …………………………………....…20

❑ Post pregnancy checkups ………………….……...21

❑ Sinus infection........................................................22

❑ Lymphoma ……………...........................................23

❑ Diabetes and liver issue …………………….……...24

❑ Swollen lymph nodes…………………………….....25

❑ Hypertension, arthritis, & orthopedic problems.......26

❑ Eye problem……………..........................................27

❑ Fibromyalgia and MS ……………..........................28

❑ Defibrillator implant ………………........................…29

❑ Liver issue………….. ……………........................…30

❑ Did not answer………………………………………..98

❑ Not applicable………………………………………...99

❑ Did not complete survey ……………………….…....99999

AS3. Has anyone in your household made an **emergency visit to a hospital emergency department** for any reason **in the last 12 months**?

❑ Yes ………………………………....1

❑ No [SKIP TO AS4]……...............0

❑ Don’t Know [SKIP TO AS4]…...8

❑ No response [SKIP TO AS4]…...9

❑ Did not complete survey ………....99999

AS3a. Approximately how many times **in the last 12 months**?

❑ 1 to 3 times………………………..1

❑ More than 3 times………………...2

❑ Don’t Know [SKIP TO AS4]……..8

❑ Not applicable…………………… 9

❑ Did not complete survey …….......99999

AS3b. Could you please explain the emergency?

❑ Broken leg ………………………………………...…1

❑ Fainted................................................................…2

❑ Cut by paper shredder………….……………......…3

❑ Knee problem ………………………………....….…4

❑ Minor cut ……………………………………….....…5

❑ Chest pain ………………………………………...…6

❑ Infection ……………….………………………......…7

❑ Allergic reaction ……………...............................…8

❑ Injury...................................................................…9

❑ Eye problem ……………………………………..…10

❑ Throat infection…………………………….….……11

❑ Baby delivery…………………………………..……12

❑ Baby sick…………………………………..……...…13

❑ Wind pipe inflammation ……………………..…..…14

❑ Concussion……………………………….…….……15

❑ Ear infection……………………………………....…16

❑ Swollen toe after cut………………………..….……17

❑ Cut eyelid ……………………………………..…...…18

❑ Wheezing & high fever………………………………19

❑ Whiplash………………………………………..….…20

❑ Afraid of Deep vein thrombosis (no diagnosis) ..…21

❑ Pregnancy …………………………….…………...…22

❑ Swollen lymph nodes ……………........................…23

❑ Migraines………………. ………………………….…24

❑ Child hurt head……..……………………………...…25

❑ Heart condition………………………………….....…26

❑ Pneumonia………………………………………....…27

❑ Multiple sclerosis & stomach problem………….......28

❑ Asthma………………………………………………...29

❑ Clogged ear…………………………………………...30

❑ Not applicable………………………………………...99

❑ Did not complete survey ……………………..……...99999

AS4. Has anyone in your household been admitted to the hospital overnight for any reason **in the last 12 months**?

❑ Yes ………………………………....1

❑ No [SKIP TO SH1]……...............0

❑ Don’t Know [SKIP TO SH1]…...8

❑ No response [SKIP TO SH1]…...9

❑ Did not complete survey ……..…..99999

AS4a. Approximately how many times **in the last 12 months**?

❑ 1 to 3 times………………………..1

❑ More than 3 times…………….......2

❑ Don’t Know [SKIP TO SH1]……..8

❑ Not applicable…………………….9

❑ Did not complete survey …..….....99999

AS4b. Could you please explain the reason for being admitted?

❑ Swine flu ……………………………………………..…1

❑ Asthma, staph infection, & allergic reaction……….…2

❑ Chest pain & surgery………….……………….….....…3

❑ Infections from surgery & blood pressure issues……4

❑ Injury……………………………………………….......…5

❑ Multiple sclerosis & stomach problem………….…..…6

❑ Child birth……………….………………………..........…7

❑ Wind pipe infection…………….................................…8

❑ Lymphoma surgery...................................................…9

❑ Back surgery………………………………………..……10

❑ Issue with pregnancy…………….………….….………11

❑ Heart condition………………………….…......…..……12

❑ Did not answer.………………………….…......…..……13

❑ Not applicable………………………..….…...….....……99

❑ Did not complete survey ……………………..………...99999

AS5. Is there something in your home that you think may have made you feel ill or negatively impacted your health?

❑ Yes…………………………………..1

❑ No [SKIP TO HS9]……………......0

❑ Not asked…………………….…......2

❑ Don’t Know [SKIP TO HS9]……...8

❑ No response [SKIP TO HS9]….....9

❑ Did not complete survey ……..…...99999

HS5a. Aspect of the home:

❑ Radiator making apartment too hot………...1

❑ Cell phone tower on roof………………….....2

❑ Bad air from car exhaust………………….....3

❑ Mold ………………………………………..…..4

❑ Secondhand smoke…………………………..5

❑ Paint peeling…………………………………..6

❑ Outdoor air pollution………………………….7

❑ Bad smells from outdoor garbage……….…..8

❑ New Paint……………………………………...9

❑ Dry Air……………………………………..…..10

❑ Noise from neighbors…………………….....11

❑ Apartment too hot and too humid……….....12

❑ Apartment too hot and lack of fresh air…....13

❑ Loud construction outside…………………..14

❑ Cockroaches………………………………….15

❑ Mold and lack of heat…………………….….16

❑ Dust……………………………………..……..17

❑ Not applicable………………………………..99

❑ Did not complete survey ……………….…...99999

HS5b. Impact on health or wellbeing:

❑ Congestion & migraines………………….....1

❑ Irritates Eczema………………………..…….2

❑ Asthma Irritated……………………………....3

❑ Feeling Sick…………………………………..4

❑ Feeling lightheaded…………………..……..5

❑ Migraines……………………………………..6

❑ Stress and lack of sleep…………………….7

❑ Irritates Allergies……………………………..8

❑ Feeling Sluggish……………………………..9

❑ Stress………………………………………...10

❑ Nosebleeds……………………………….....11

❑ Cold and flu………………………………....12

❑ Lack of sleep and coughing……………….13

❑ Lack of sleep………………………………...14

❑ Don’t Know…………………………………..88

❑ Not applicable……………………………….99

❑ Did not complete survey ……………….......99999

SH1. What is the main equipment you use you heat your home? (SELECT ONLY ONE)

❑ No heating equipment used………………….…………………...……0

❑ Heat pump ………………………………………………………...…..…1

❑ Central warm-air furnace with ducts to individual rooms other than a heat pump…………………………………………………...…………………..…..2

❑ Steam/hot water system with radiators/convectors in each room or pipes in the floor or walls ……………………………………………………………..……3

❑ Built-in electric units in each room installed in walls, ceiling, baseboard, or floor……………………………………………………............................……4

❑ Built-in floor/wall pipeless furnace……………….………………….....5

❑ Built-in room heater burning gas, oil, or kerosene……………………6

❑ Heating stove burning wood, coal, or coke……………………………7

❑ Portable electric heaters………………………………………….……...8

❑ Portable kerosene heaters…………………………………….………...9

❑ Fireplace…………………………………………………………………..10

❑ Cooking stove that is used to heat your home as well as to cook…..11

❑ Some other equipment (Specify __________________)……………12

❑ Don’t Know…………………………………………………………..……88

❑ Did not complete survey ………………………………………...….…...99999

SH2. Do you use any other types of equipment to heat your home (space heater, stove), or take any other steps to deal with cold temperatures in your apartment (such as wear extra layers of clothing or complain to building management)?

❑ Yes…………….……………….….1

❑ No [SKIP TO SH3]…...………...0

❑ Don’t Know [SKIP TO SH3].…..8

❑ Did not complete survey ……......99999

SH2a. What is the secondary equipment or other methods you use to stay warm?

❑ Extra clothing ………………………………………….1

❑ More blankets at night………………………………...2

❑ Steam/hot water system with radiators/convectors in each room or pipes

in the floor or walls ……………………………………....…3

❑ Built-in electric units in each room installed in walls, ceiling, baseboard,

or floor…………………………………………………..……4

❑ Built-in floor/wall pipeless furnace………………..….5

❑ Built-in room heater burning gas, oil, or kerosene…6

❑ Heating stove burning wood, coal, or coke…………7

❑ Portable electric heaters………………………….…...8

❑ Portable kerosene heaters…………………………....9

❑ Fireplace………………………………………………..10

❑ Cooking stove that is used to heat your home ……..11

❑ Floorboard heating………………………… …………12

❑ More blankets at night & cooking stove…………..…28

❑ Extra clothing, more blankets at night & portable electric heater……………………………………………………..…128

❑ Portable electric heaters & cooking stove………..…811

❑ Don’t Know…………………………………………..…88

❑ No Response………….………………………….....…99

❑ Did not complete survey ………….……………...…...99999

SH3. During the winter, do you usually adjust the temperature differently depending on whether anyone is home, or during sleeping hours?

❑ Yes………………………….....1

❑ No………………………….…..0

❑ Don’t Know……………….…..8

❑ Did not complete survey …....99999

SH4. Last winter, for any reason, was your housing unit so cold for 24 hours or more that it was uncomfortable?

❑ Yes…………………………………………………...1

❑ No …………………………………………………....0

❑ Did not live here last winter [SKIP TO AC1]...…..2

❑ Don’t Know…………………………………………8

❑ No Response……………………………………….9

❑ Did not complete survey …………………….…....99999

SH5. Do you ever open windows because your housing unit was too hot?

❑ Yes………………………………………………..1

❑ No [SKIP TO UB1].……………………………...0

❑ Did not live here last winter [SKIP TO AC1]….2

❑ Don’t Know [SKIP TO AC1].……………………8

❑ Did not complete survey ………………………..99999

SH5A. About how often did you open windows because it was too hot?

❑ Every day……………………....1

❑ 2 to 5 times a week.………......2

❑ Once a week…………………..3

❑ Less than once a week.………4

❑ Don’t Know…………………….8

❑ Not applicable.………………...9

❑ Did not complete survey …......99999

Air Conditioning:

AC1. Do you have air conditioning equipment in your home?

❑ Yes…………………………..…....1

❑ No [SKIP TO AC6]………….....0

❑ Don’t Know [SKIP TO AC6]…...8

❑ Did not complete survey …….....99999

AC2. What kind of air-conditioning equipment does your home have?

❑ A central system…………………………………………….....1

❑ Individual units in the windows or wall [SKIP TO AC3]……2

❑ Both central and individual units………………………….....3

❑ Don’t Know [SKIP TO AC3]……………………………...…..8

❑ Not applicable………….………………………………………9

❑ Did not complete survey ……………………………………...99999

AC3. How many air conditioning units do you have in your home?

Number of AC units: ________

❑ Not applicable………….………..…99

❑ Did not complete survey ……….....99999

AC4. Thinking about summer, does the air-conditioning system in your home cool all of the rooms or only some of the rooms in your home?

❑ None of the rooms cooled……..0

❑ All of the rooms…………………1

❑ Only some of the rooms……….2

❑ Don’t Know……………………..8

❑ Not applicable………….………9

❑ Did not complete survey ……...99999

AC5. Which of these statements best describes the way your household uses the air-conditioning system during the summer?

❑ Not used at all……………………………………………………....0

❑ Turned on only a few days or nights when really needed……..1

❑ Turned on quite a bit…………………………………………….....2

❑ Turned on just about all summer………………………………....3

❑ Don’t Know……………………………………………………….....8

❑ Not applicable………….……………………………………...…....9

❑ Did not complete survey …………………………………………..99999

AC6: Do you use any of the following alternative cooling methods? (*Check all that apply*)

❑ No………………………………………………………..…...….0

❑ Electric fans………………………………………………….....1

❑ Opening windows………………………………………...…....2

❑ Electric fans & opening windows..........................................12

❑ Electric fans & opening windows & lightweight clothes.......123

❑ Electric fans & opening windows & cold showers……….....124

❑ Electric fans & opening windows & ice packs…………….....125

❑ Don’t Know……………………………………………………...8

❑ Not asked……………………………………………………......9

❑ Did not complete survey ……………………………….……...99999

Energy Assistance:

EA1. In the past 12 months have you experienced any hardship with paying for utilities?

❑ Yes…………………………....1

❑ No [SKIP TO HQ1]……….....0

❑ Don’t Know………………......8

❑ Was not asked …………...…9

❑ Did not complete survey …...99999

For the last 12 months…

EA2. How often did you worry that you wouldn’t be able to pay your home energy bill?

❑ Almost every month………....3

❑ Some months ………………..2

❑ Only 1 or 2 months…………..1

❑ Never………………………….0

❑ Not applicable …………….…99

❑ Did not complete survey …....99999

EA3. If some or all of the gas or electricity used in your home was paid for in some other way, who paid for that? Was it…

❑ None……………………………………0

❑ A relative………………………………..1

❑ A rental or condominium agent ……...2

❑ LIHEAP ………………………………...3

❑ Don’t know ……………………..……….8

❑ Was not asked ……………………...…88

❑ Not applicable ………………..……..…99

❑ Did not complete survey ………….......99999

EA4. Have you ever used a payment plan with Con Edison in order to pay your utility expenses?

❑ Yes…………………………….1

❑ No [SKIP TO EA5]…………...0

❑ Don’t Know [SKIP TO EA5]....8

❑ Was not asked ………………88

❑ Not applicable …………….…99

❑ Did not complete survey …....99999

EA4a. Do you currently have a payment plan with Con Edison in order to pay your utility expenses?

❑ Yes…………………………….1

❑ No ……………………………..0

❑ Don’t Know ………………..…8

❑ Was not asked .……………...88

❑ Not applicable …………….…99

❑ Did not complete survey …....99999

EA5. How often did you reduce your expenses for what you consider to be basic household necessities?

❑ Almost every month………....3

❑ Some months ………………..2

❑ Only 1 or 2 months…………..1

❑ Never………………………….0

❑ Not applicable …………….…99

❑ Did not complete survey …....99999

EA6. How often did you have a supplier of your electric or home heating service threaten to disconnect your electricity or home heating fuel service?

❑ Almost every month………....3

❑ Some months ………………..2

❑ Only 1 or 2 months…………..1

❑ Never………………………….0

❑ Not applicable …………….…99

❑ Did not complete survey …....99999

**The next few questions are about thermal comfort in your home**

HQ1. Overall, would you say that your home is…

❑ Well insulated……………………………1

❑ Adequately insulated……………………2

❑ Poorly insulated………………………….3

❑ No insulation [IF VOLUNTEERED]…....0

❑ Don’t Know……………………………….8

❑ Did not complete survey ……………......99999

HQ2. How often do you or other members of your household find your home too drafty during the winter? Would you say it is. . .

❑ All of the time………………....3

❑ Most of the time …………..….2

❑ Some of the time…………..…1

❑ Never…………………….……0

❑ Did not complete survey …....99999

HQ3. In the past 12 months, how often did you close off part of your home because it was uncomfortably hot or cold?

❑ Almost every month………....3

❑ Some months ………………..2

❑ Only 1 or 2 months…………..1

❑ Never………………………….0

❑ Did not complete survey …....99999

HQ4. In the past 12 months, how often was your home at a temperature that you felt was uncomfortable at any time of the year?

❑ Almost every month………....3

❑ Some months ………………..2

❑ Only 1 or 2 months…………..1

❑ Never………………………….0

❑ Was not asked ………………88

❑ Did not complete survey …....99999

HQ5. In the past 12 months, how often did you leave your home for part of the day because it was too hot or too cold?

❑ Almost every month………....3

❑ Some months ………………..2

❑ Only 1 or 2 months…………..1

❑ Never………………………….0

❑ Did not complete survey …....99999

HQ6. Do you have a stove that has both burners and an oven?

❑ Yes…………………………...1

❑ No ……………………………0

❑ Did not complete survey …..99999

HQ7. What fuel does your stove use?

❑ Electricity ……………………………………1

❑ Natural gas from underground pipes…….2

❑ Propane (bottled gas) ……………………..3

❑ Did not complete survey ………………....99999

HQ8. In the past 12 months, how often did you use your kitchen stove or oven to provide heat?

❑ Almost every month………....3

❑ Some months ………………..2

❑ Only 1 or 2 months…………..1

❑ Never………………………….0

❑ Did not complete survey …...99999

HQ9. Do you ever add moisture to the air in your home/apartment?

❑ Yes…………………………………....1

❑ No [SKIP TO KA1]…………………..0

❑ Did not complete survey …………...99999

HQ9a. If “Yes”, what method has been used? (*Select ALL that apply*)

❑ Cool Mist Humidifier……………………………….……1

❑ Hot Mist Humidifier………………………………….…..2

❑ Pans of Water on Radiators………………………….....3

❑ Boiling Water on Stove……………………………..…...4

❑ Cool Mist Humidifier & Pans of water on radiator……13

❑ Don’t Know……………………………………….……....8

❑ Not applicable……………………………………………9

❑ Did not complete survey ………………………….…....99999

KA1. Which of the categories shown best describes, on average, how often you use your oven?

❑ More than once a day……………………….5

❑ Once a day…………………………………...4

❑ Between once a day and once a week……3

❑ Once a week…………………………………2

❑ Less than once a week……………………..1

❑ Never…………………...……………………..0

❑ Don’t Know…………………………………..8

❑ Did not complete survey ……………….......99999

KA2. Does anyone in your household use any other equipment to do any cooking?

❑ Yes……………………………..…..1

❑ No [Skip to KA3]………………...0

❑ Don’t Know [Skip to KA3]………8

❑ No Response [Skip to KA3]……9

❑ Did not complete survey …….......99999

KA2a. Which other equipment is used in your household to do any cooking? (*Check all that apply*)

❑ Microwave…………………………...…….1

❑ Hot plate………………………………...…2

❑ Toaster …………………………………….3

❑ Deep Fryer…………………......................4

❑ Microwave & hot plate……………...........12

❑ Microwave & toaster………………….......13

❑ Microwave & broiler………….……….......14

❑ Toaster & blowtorch………………….......35

❑ Microwave, hot plate & toaster …............123

❑ Microwave, toaster, & Deep Fryer...........134

❑ Microwave, toaster, & Crock pot……......135

❑ Microwave, toaster, & Foreman grill…...136

❑ Don’t Know……………….………………...8

❑ Not applicable……………….…………….9

❑ Did not complete survey …………….…...99999

KA3. In the past 12 months has there been a fire in your home?

❑ Yes……………………………..1

❑ No [SKIP TO KA4]…………..0

❑ Did not complete survey …....99999

KA3a. In the past 12 months did any fire start in your home as a result of using an alternate *heating* source, such as space heaters, electric blankets, your kitchen stove or oven, heating stove, furnace, or your fireplace?

❑ Yes……………………………1

❑ No …………………………….0

❑ Not applicable.………………9

❑ Did not complete survey …...99999

KA3b. In the past 12 months, how many individuals living in your home needed medical attention because of fire in the home?

Number individuals needing medical attention: ____________

❑ Not applicable.……………….9

❑ Did not complete survey …....99999

KA4. Has your home been renovated or had any repairs done in the last 12 months?

❑ Yes………………………………….1

❑ No [SKIP TO KA1]………………..0

❑ Don’t Know [SKIP TO KA1]……...8

❑ No response [SKIP TO KA1]……9

❑ Did not complete survey ………...99999

KA4a. If “Yes”, what type of repairs/renovations occurred in the home/apartment in the last 12 months? *(circle ALL that apply)*

❑ Leaky pipes…………………………………………………...1

❑ Holes/Cracks in the Ceiling/Wall…………………………...2

❑ Refinishing floors…………………………………………….3

❑ Painting……………………………………………………….4

❑ Plaster repair………………………………………………….5

❑ Sink replacement…………………………………………….6

❑ Oven repair & leak in the ceiling…..……………………….7

❑ New Cabinets……………………………………………..….8

❑ Toilet repair………………………………………………..….9

❑ Window repair……………………………………………….10

❑ Leaky pipes & painting.…………………………………….14

❑ Holes/Cracks in the Ceiling/Wall & Refinishing floors ….23

❑ Holes/Cracks in the Ceiling/Wall & painting…………..….24

❑ Refinishing floors & painting………………..…………..….34

❑ Leaky pipes, refinishing floors & painting………………...134

❑ Refinishing floors, painting, & insulation……………...…..345

❑ Leaky pipes, Holes/Cracks in the Ceiling/Wall, Refinishing floors, painting, & roof repair …………………………….…………..…12345

❑ Not applicable….……………………………………………..99

❑ Did not complete survey …………………………….……....99999

Windows:

WN1. Approximately, how many windows does your home/apartment have? Each window that opens separately should be counted as one window. Leave out of your count any windows that are in unheated parts of your home/apartment.

❑ 1 or 2 …………………………………….…..1

❑ 3 to 5………………………….......................2

❑ 6 to 9 ………………………………………...3

❑ 10 to 15……………………….......................4

❑ 16 to 19……………………….......................5

❑ Don’t Know…………………………………..8

❑ Did not complete survey …………….……..99999

WN2. Which best describes the type of glass in the windows of your home/apartment? Do not consider storm windows.

❑ Single-pane glass…………………………....1

❑ Double-pane glass…………………………..2

❑ Double-pane glass with Low-e coating…....3

❑ Triple-pane glass………………………….....4

❑ Triple-pane glass with Low-e coating……...5

❑ Don’t Know………………………………..….8

❑ No Response………….…………………..…9

❑ Did not complete survey …………………....99999

SE1. Now I would like to ask you a bit about the quality of the housing you live in now.

Does your housing have…

SE1a. Broken or damaged windows?

❑ Yes……………………………..1

❑ No………………………………0

❑ Don’t Know……………………8

❑ No response…………………..9

❑ Did not complete survey ….....99999

SE1b. Broken, stopped up or overflowing toilets?

❑ Yes………………………...…..1

❑ No…………………………...…0

❑ Don’t Know………………...…8

❑ No response……………….....9

❑ Did not complete survey …....99999

SE1c. Other busted plumbing?

❑ Yes………………………..…..1

❑ No…………………………..…0

❑ Don’t Know………………..…8

❑ No response………………....9

❑ Did not complete survey …...99999

SE2. On maintenance of the building, are you…

❑ Completely satisfied………..…….3

❑ Partly satisfied………………..…...2

❑ Dissatisfied…………………..…….1

❑ Don’t Know…………………..…….8

❑ No Response……………..……….9

❑ Did not complete survey ………....99999

SE3. In the INSIDE walls or ceilings of this housing unit,are there any OPEN HOLES or CRACKS WIDER THAN THE EDGE OF A DIME?

❑ Yes………………………..…...1

❑ No ………………………..……0

❑ Don’t Know………………..….8

❑ No Response……………...….9

❑ Did not complete survey ….....99999

Pests/Mold

PM1. Is your home infested with cockroaches, and if yes, how infested would you say your home is?

❑ No, not infested [SKIP to PM2]….….0

❑ Yes extremely infested…………..….4

❑ Yes very infested…………...………..3

❑ Yes somewhat infested……..………2

❑ Yes, hardly infested………..………...1

❑ Don’t know……………………......…..8

❑ Did not answer……………...………..9

❑ Was not asked……………...….……..99

❑ Did not complete survey …….….…..99999

PM1a. Have you done anything about the cockroaches, and if yes, what have you done? (CHECK ALL THAT APPLY).

❑ No, nothing………………………………………………….0

❑ Yes, used insecticides, bug sprays, or poison…………..1

❑ Yes, hired an exterminator or other professional……….2

❑ Yes, kill them by hand………………………………………3

❑ Yes, used insecticides, bug sprays, or poison & hired an exterminator or other professional ………………………………………..…..12

❑ Don’t Know………………………………………………….8

❑ Not applicable……………………………………………….9

❑ Was not asked……………...................................………..99

❑ Did not complete survey ……………………………….......99999

PM2. Is your home infested with other insects (such as ants or beetles) or spiders, and if yes, how infested would you say your home is?

❑ No, not infested [SKIP to PM3]……..0

❑ Yes extremely infested…………..….4

❑ Yes very infested…………...………..3

❑ Yes somewhat infested……..………2

❑ Yes, hardly infested………..………...1

❑ Don’t know……………………......…..8

❑ No Response………………..………..9

❑ Was not asked……………...………..99

❑ Did not complete survey ……….…...99999

PM2a. Have you done anything about the other insects or spiders, and if yes, what have you done? (CHECK ALL THAT APPLY)

❑ No, nothing………………………………………………….0

❑ Yes, used insecticides, bug sprays, or poison…………..1

❑ Yes, hired an exterminator or other professional……….2

❑ Yes, kill them by hand………………………….………..…3

❑ Yes, sealed cracks….………………………………………4

❑ Yes, used insecticides, bug sprays, or poison & hired an exterminator or other professional ………………………………………..…..12

❑ Don’t Know………………………………………….……….8

❑ Not applicable……………………………………………….9

❑ Was not asked……………...………………………………..99

❑ Did not complete survey ……………………………….......99999

PM3. Is your home infested with rats or mice, and if yes, how infested would you say your home is?

❑ No, not infested [SKIP to PM4]……..0

❑ Yes extremely infested…………..….4

❑ Yes very infested…………...………..3

❑ Yes somewhat infested……..………2

❑ Yes, hardly infested………..………...1

❑ Don’t know……………………......…..8

❑ No Response………………..………..9

❑ Was not asked……………...………..99

❑ Did not complete survey …………....99999

PM3a. Have you done anything about the rats or mice, and if yes, what have you done? (CHECK ALL THAT APPLY).

❑ No nothing…………………………………………………………….....0

❑ Yes, used bait or poison……………………………………………......1

❑ Yes, hired an exterminator or other professional…………………….2

❑ Yes, clog holes……………………………………………………….….3

❑ Yes, non-poison traps……………………………………………….….4

❑ Yes, bought a cat…………………………………………………….….5

❑ Yes, used insecticides, bug sprays, or poison & hired an exterminator or other professional ……………………………………………………….…..12

❑ Yes, used bait or poison & clog holes……………………………...…13

❑ Yes, hired an exterminator or other professional & clog holes…….23

❑ Don’t Know……………………………………………………………….8

❑ Not applicable…………………………………………………………....9

❑ Was not asked……………………………………………….....………..99

❑ Did not complete survey …………………………………………..…....99999

PM4. Does your home frequently have a mildew odor or musty smell?

❑ Yes……………………………1

❑ No …………………………….0

❑ Don’t Know…………………..8

❑ Was not asked………………..99

❑ Did not complete survey …...99999

PM5. Have you seen mold in your home in the past 12 months?

❑ Yes……………………………..1

❑ No [Skip to T1]…………….….0

❑ Don’t Know [Skip to T1]……..8

❑ No response [Skip to T1]……9

❑ Was not asked………………..99

❑ Did not complete survey …...99999

PM5a. Have you done anything about the mold, and if yes, what have you done? (CHECK ALL THAT APPLY).

❑ No nothing…………………………………………………………………....0

❑ Yes, cleaned with bleach………………………………………………..….1

❑ Yes, cleaned with other chemical mold remover………………………....2

❑ Yes, cleaned with natural mold remover (vinegar or peroxide)……........3

❑ Yes air Conditioned……………………………………………………….....4

❑ Yes, ventilation (fans)……………………………………………………….5

❑ Yes, used a dehumidifier…………………………………………………....6

❑ Yes contacted a Professional……………………………………………....7

❑ Yes, cleaned with bleach & contacted a professional…...……………...17

❑ Other. Please Specify _________...........................................................8

❑ Don’t know…………………………………………………………………....88

❑ Not applicable…………………………………………………………….….99

❑ Was not asked……………...……………………………………………..….999

❑ Did not complete survey ……………………………………………….…...99999

Neighborhood Quality:

NQ1. How long have you lived in your neighborhood?

NQ1a. Record # of years _____ _____

❑ Was not asked……………...……………………………………….999

❑ Did not complete survey ………………………………………......99999

NQ1b. Record # of months _____ _____

❑ Was not asked……………...……………………………………….999

❑ Did not complete survey …………………………………………..99999

NQ2. Please tell me how often these things happen in your neighborhood *(Choose* ***ONE****)*

NQ2. Houses and buildings are kept in good repair on the outside

❑ Very often ……………….……4

❑ Fairly often …………………....3

❑ Sometimes………………….…2

❑ Almost never……………….…1

❑ Never……………………….….0

❑ Don’t Know…………………....8

❑ No response …………….……9

❑ Was not asked……………….99

❑ Did not complete survey …....99999

NQ2a. The neighborhood streets are busy with shoppers, mothers with strollers, and children playing

❑ Very often ……………….……4

❑ Fairly often ……………….…...3

❑ Sometimes……………….……2

❑ Almost never…………….……1

❑ Never…………………….…….0

❑ Don’t Know…………………...8

❑ No response ……………….…9

❑ Was not asked …………….…99

❑ Did not complete survey ….....99999

NQ2b. Homeless or unemployed people are hanging around

❑ Very often ……………….……4

❑ Fairly often …………………...3

❑ Sometimes……………………2

❑ Almost never…………………1

❑ Never………………………….0

❑ Don’t Know…………………...8

❑ No response …………………9

❑ Was not asked ………………99

❑ Did not complete survey …....99999

NQ2c. There are vacant lots or boarded up buildings

❑ Very often …………………4

❑ Fairly often ………………...3

❑ Sometimes…………………2

❑ Almost never………………1

❑ Never……………………….0

❑ Don’t Know………………...8

❑ No response ………………9

❑ Was not asked ……………99

❑ Did not complete survey ....99999

NQ3. How worried are you about being the victim of a crime in your neighborhood? *(Select One)*

❑ Very worried …………………4

❑ Fairly worried ………………..3

❑ Somewhat worried……….….2

❑ A little worried………………..1

❑ Not at all worried………….....0

❑ Don’t Know…………………..8

❑ No response……………….…9

❑ Was not asked ………..…..…99

❑ Did not complete survey …....99999

NQ4. I am going to read several statements about your neighborhood. As I read each one, please answer yes or no.

NQ4. My neighborhood is a good place to live.

❑ Yes………………………….…1

❑ No ………………………….….0

❑ Don’t Know…………………....8

❑ No response………………..…9

❑ Was not asked ……………….99

❑ Did not complete survey ….....99999

NQ4a. People move in and out of my neighborhood a lot.

❑ Yes………………………….…1

❑ No ………………………….….0

❑ Don’t Know……………….…..8

❑ No response……………….…9

❑ Was not asked ………………99

❑ Did not complete survey …....99999

NQ5. How often do you avoid going outside, or avoid letting your children go outside, for safety reasons? *(Choose* ***ONE****)*

❑ Never……………………….....0

❑ Rarely ………………………...1

❑ Sometimes……………………2

❑ Often…………………………..3

❑ All the time……………………4

❑ Don’t Know…………………...8

❑ No response ………………….9

❑ Was not asked ………………99

❑ Did not complete survey …....99999

NQ6. Thinking of the area where you live, in the past 12 months, have members of your household had regular access to public transportation?

❑ Yes………………………….…1

❑ No ………………………….….0

❑ Don’t Know……………….…..8

❑ No response………………..…9

❑ Was not asked …………….…99

❑ Did not complete survey ….....99999

Tempavg.

Average indoor temperature _________ ^o^F

❑ Missing Data …………………………….999

HiTemp.

High Temperature _________ ^o^F

❑ Missing Data …………………………….999

LoTemp.

Low Temperature _________ ^o^F

❑ Missing Data …………………………….999

Hmdavg.

Average indoor humidity _________ %

❑ Missing Data …………………………….999

HmdHi.

High Humidity level _________ %

❑ Missing Data …………………………….999

HmdLo.

Low Humidity level _________ %

❑ Missing Data …………………………….999

InFltr. Indoor Filter Net Mass (mg) ____________mg.

❑ Missing Data ………………………………….…….999

OutFltr. Outdoor Filter Net Mass (mg)__________mg.

❑ Missing Data ………………………………….…….999

INBC. Indoor Black Carbon ____________ng/mm2.

❑ Missing Data ………………………………….…….999

INSHS. Indoor Secondhand Smoke ____________ng/mm2.

❑ Missing Data ………………………………………….…….999

INSit. Indoor SIT___________________ng/mm2

❑ Missing Data ………………………………….…….999

OUTBC. Outdoor Black Carbon ____________ng/mm2.

❑ Missing Data ………………………………….….…….999

OUTSHS. Outdoor Secondhand Smoke ____________ng/mm2.

❑ Missing Data ……………………………………………….…….999

OUTSit. Outdoor SIT___________________ng/mm2

❑ Missing Data ………………………………….…….999
